# Recent advances in recording and modulation technologies for next-generation neural interfaces

**DOI:** 10.1016/j.isci.2021.103550

**Published:** 2021-12-03

**Authors:** Ji-Won Hong, Chanwoong Yoon, Kyunghyun Jo, Joon Hee Won, Seongjun Park

**Affiliations:** 1 Program of Brain and Cognitive Engineering, Korea Advanced Institute of Science and Technology (KAIST), Daejeon 34141, Korea; 2 Department of Bio and Brain Engineering, Korea Advanced Institute of Science and Technology (KAIST), Daejeon 34141, Korea; 3 KAIST Institute of Health Science and Technology (KIHST), Korea Advanced Institute of Science and Technology (KAIST), Daejeon 34141, Korea

**Keywords:** Neuroscience, Techniques in neuroscience, Biotechnology, Bioelectronics

## Abstract

Along with the advancement in neural engineering techniques, unprecedented progress in the development of neural interfaces has been made over the past few decades. However, despite these achievements, there is still room for further improvements especially toward the possibility of monitoring and modulating neural activities with high resolution and specificity in our daily lives. In an effort of taking a step toward the next-generation neural interfaces, we want to highlight the recent progress in neural technologies. We will cover a wide scope of such developments ranging from novel platforms for highly specific recording and modulation to system integration for practical applications of novel interfaces.

## Graphical abstract

**Figure:**
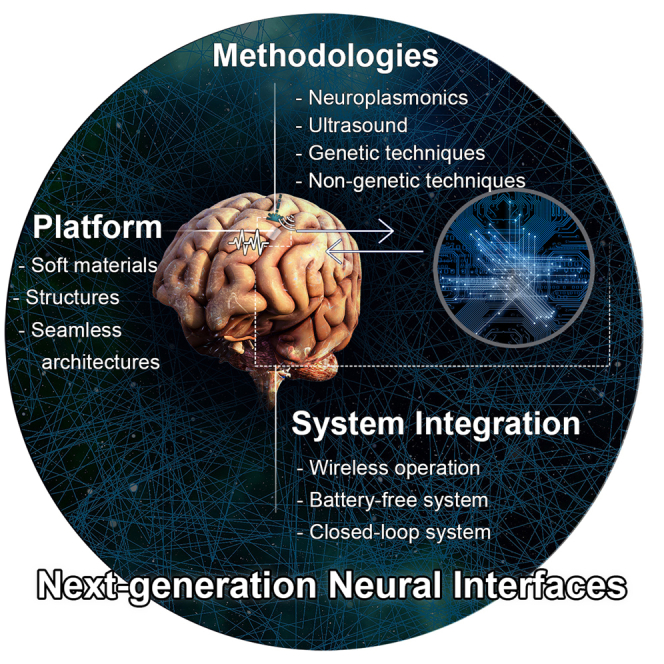

## Introduction

The dynamic interactions of neural activities generated from the neural system are the underlying driving force of perception, cognition, and behavior of human beings. Thus neuroscientists and neural engineers are striving to decipher the meaning of the complex patterns to apply them to curing neurological diseases or restoring functions of the body. To achieve this goal, the ability to interact simultaneously with multiple neurons lying deep inside our neural system is crucial. For the last few decades, significant progress has been made in developing neural interfaces tasked with interacting with the dynamic neural system. One of the pioneering researches in the 1950s by Hubel et al. was the observation of neural activities in the mammalian (cat) brain using tungsten microwire electrodes (Hubel, 1957). His work had motivated many researchers to dive into neuroscience studies (Desimone et al., 1984; Hubel and Wiesel, 1962; O'Keefe and Dostrovsky, 1971) and to develop different probes with enhanced performance (McNaughton et al., 1983). Assisted by the advances in microfabrication techniques, myriads of devices with diverse layouts have been proposed including densely organized electrode arrays based on doped silicon (known as Utah arrays) (Campbell et al., 1991; Maynard et al., 1997) and silicon shanks mounting longitudinal sets of electrodes (known as Michigan probes) (Wise et al., 1970). Concurrently, methodological investigations to incorporate a variety of signaling modalities have brought notable advances, expanding the breadth of options to collect and modulate neural activities. Electrical interfacing techniques have been established as robust tools for collecting neural activities from single-neuron level to populations of neurons *in vivo* (Buzsáki, 2004; Logothetis et al., 2001). Combined with the progress in genetic engineering, especially the advent of optogenetics enabled the reliable sustained control of specific types of neurons with high precision in a short time (Boyden et al., 2005).

Despite the remarkable progress in the past decades, the ability to monitor and modulate the dynamics of the neural system is still insufficient to clearly interpret the signals and manipulate them throughout a prolonged period. First of all, conventional neural interfaces still lack chronic recording stability *in vivo*. The primary reason stems from the soft and complex nature of the neural tissues, as the mechanical mismatch between the biotic and rigid synthetic interfaces easily induces foreign body responses. Such biological responses are often responsible for the degradation of device performances, and the damage caused can even trigger neurodegeneration (Sung et al., 2020). Intimate interfacing with the target is another task that has to be solved to reach for high-fidelity signal acquisition and delivery (Li et al., 2021). Typical interfaces were composed of mechanically incompliant inorganic materials, which do not conform to a given surface or volume, attributing to cause a huge degree of unwanted noises. The second set of challenges lie in the methodological approaches, which exist both in recording as well as the stimulation. Current recording techniques generally rely on electrical pathways to transfer the signal, which is susceptible to electrical noises from surroundings. Because the electrical noises significantly disturb the sensitivity, achieving fine signals from the target region with high sensitivity is not yet an easy feat. In the stimulation approach, the emerging techniques that are expected to enable precise and cell-type specific modulation such as optogenetics also need further elaborations (Yang and Park, 2021). Visible light is highly diffusive in biological tissue, which mandates invasive methods to deliver the stimulation to the target region. Furthermore, the use of genetic modification tools is considered empirical, disturbing the clinical translation. This raises the need for the development of specific, but nongenetic, modulation techniques. Finally, in the application aspect, unprecedented developments of neural interfaces have opened up the world of brain-computer interfaces (BCIs). Some of these are for rehabilitation or restoration aids as well as exploration of fundamental neuroscience. However, there are some bottlenecks that restrict the practical application of neural interfaces. Current systems require wired connections and/or battery-powered interfaces for data transmission and power supply during operation. It results in several constraints such as discomfort in the motion of subjects because of tension of wires or weight of batteries, aggravation of scars, and restriction on the site or animal type to which the interface is to be applied.

**Figure 1:**
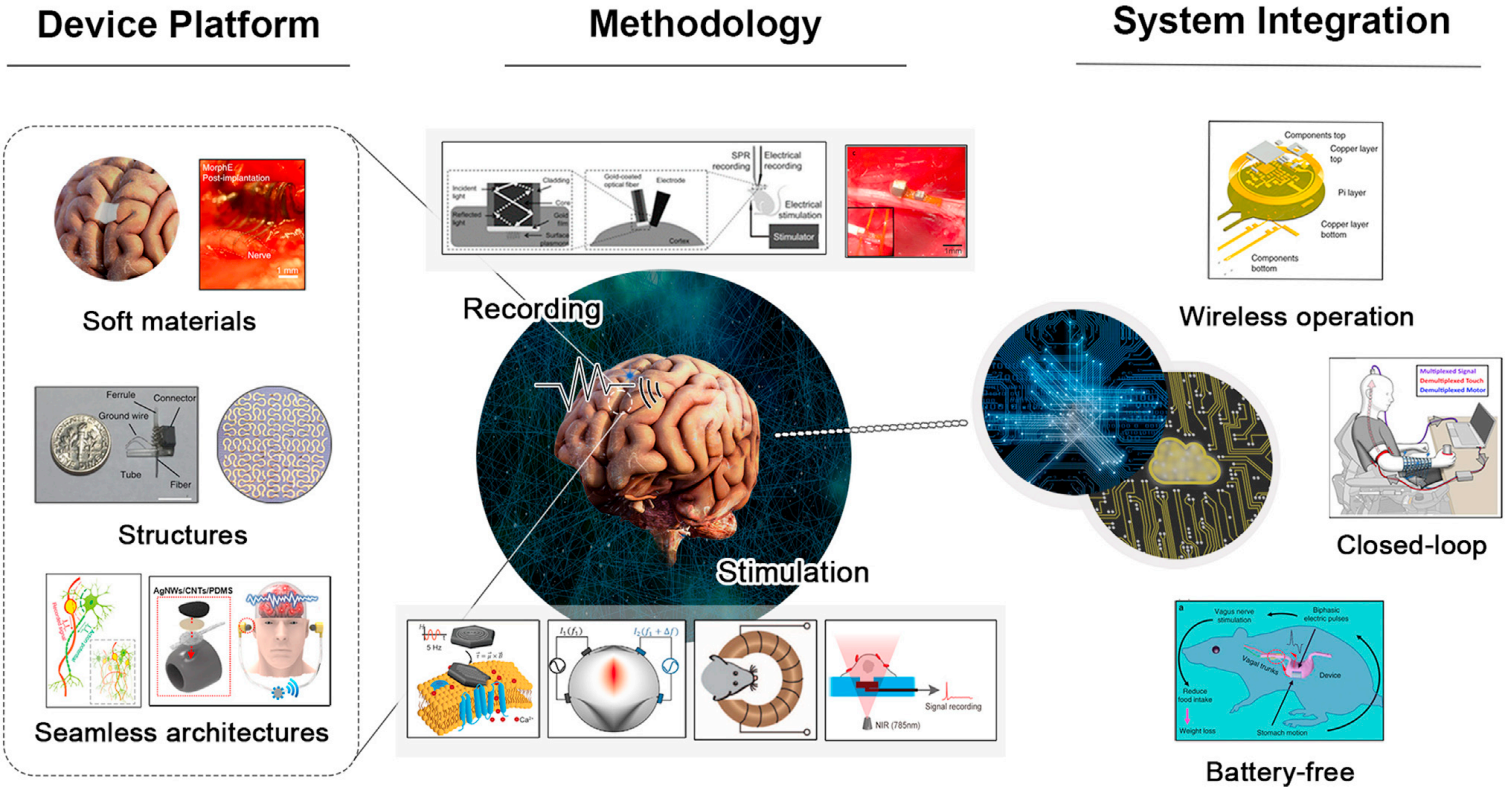
Overview of recent advances in recording and modulation technologies for next-generation neural interfaces (Device platform) Soft materials; Reproduced with permission (Liu et al., 2020), Copyright, Springer Nature. Structures; Reproduced with permission (Park et al., 2017), Copyright, Springer Nature. Reproduced with permission (Tian et al., 2019), Copyright, Springer Nature. Seamless architectures; Reproduced with permission (Yang et al., 2019), Copyright, Springer Nature. Reproduced with permission (Lee et al., 2018a), Copyright, American Chemical Society. (Methodology) Recording; Reproduced with (Kim et al., 2012), Copyright, OSA. Reproduced with (Seo et al., 2016), Copyright, Elsevier. Stimulation; Reproduced with permission (Gregurec et al., 2020), Copyright, American Chemical Society. Reproduced with permission (Grossman et al., 2017), Copyright, Elsevier. Reproduced with permission (Chen et al., 2015), Copyright AAAS. Reproduced with permission (Yoo et al., 2014), Copyright, American Chemical Society. (System integration) Wireless operation; Reproduced with permission (Gutruf et al., 2018), Copyright, Springer Nature. Closed-loop; Reproduced with permission (Ganzer et al., 2020), Copyright, Elsevier. Battery-free; Reproduced with permission (Yao et al., 2018), Copyright, Springer Nature.

In the past few years, remarkable progress on neural interfaces has been made to effectively address such challenges, as summarized by several excellent reviews (Chen et al., 2017; Lacour et al., 2016; Sung et al., 2020; Vázquez-Guardado et al., 2020; Won et al., 2020). To meet the necessary challenges and follow-up with the rapid progress, there have been needs for the systemic summary that encompasses the state-of-the-art neural recording and modulation technologies. In this review, we aim to provide a comprehensive overview of the latest advances in neural interfaces to identify and chase the emerging trends in neural interfaces and relevant technologies within a broader scope. Especially, these three categorized topics will be discussed: device platforms, methodologies, and system integration (Figure 1). In the first part, the advances in developing long-term reliable neural platforms will be presented in terms of materials, structures, and architectures. Recent efforts to design and manufacture the compliant neural devices with flexible or conformable and bio-resorbable characteristics will also be dealt with here. Next, we describe the emerging neural methodologies in detail. Novel approaches such as the use of neuroplasmonics, new genetic tools, and various nanoparticles, which have rarely been covered in previous review papers, will be introduced. Lastly, strategies and examples of the system integration for practical use of neural interfaces will be covered. In the final discussion, we will share our viewpoints on challenges and perspectives for developing next-generation neural interfaces.

## Advances in neural device platforms

Recently, a lot of effort has been made toward designing and manufacturing more advanced neural device platforms. One of the largest issues being tackled is resolving the mechanical mismatch between the biological tissue and the device, which causes acute and chronic inflammation. Such immune responses in turn significantly restrict the long-term stability and performance of the neural devices. To address these challenges, compliant device platforms have been proposed as a key for the complete integration of devices with the biosystem. Here, in order to successfully be applied in chronic usage situations while maintaining reliable performance, these compliant devices must satisfy some tough criteria such as flexibility, conformability, and bioresorbability. In this section, the design considerations and the consequent fabrication strategies for developing neural interfaces that aim to meet these criteria will be discussed.

### Toward the soft materials-based neural devices

As a step toward overcoming the limitations discussed earlier, materialistic approaches that allow the production of devices that can mimic and conform to the dynamic mechanical nature of biological systems have drawn attention with its recent progress.

**Figure 2:**
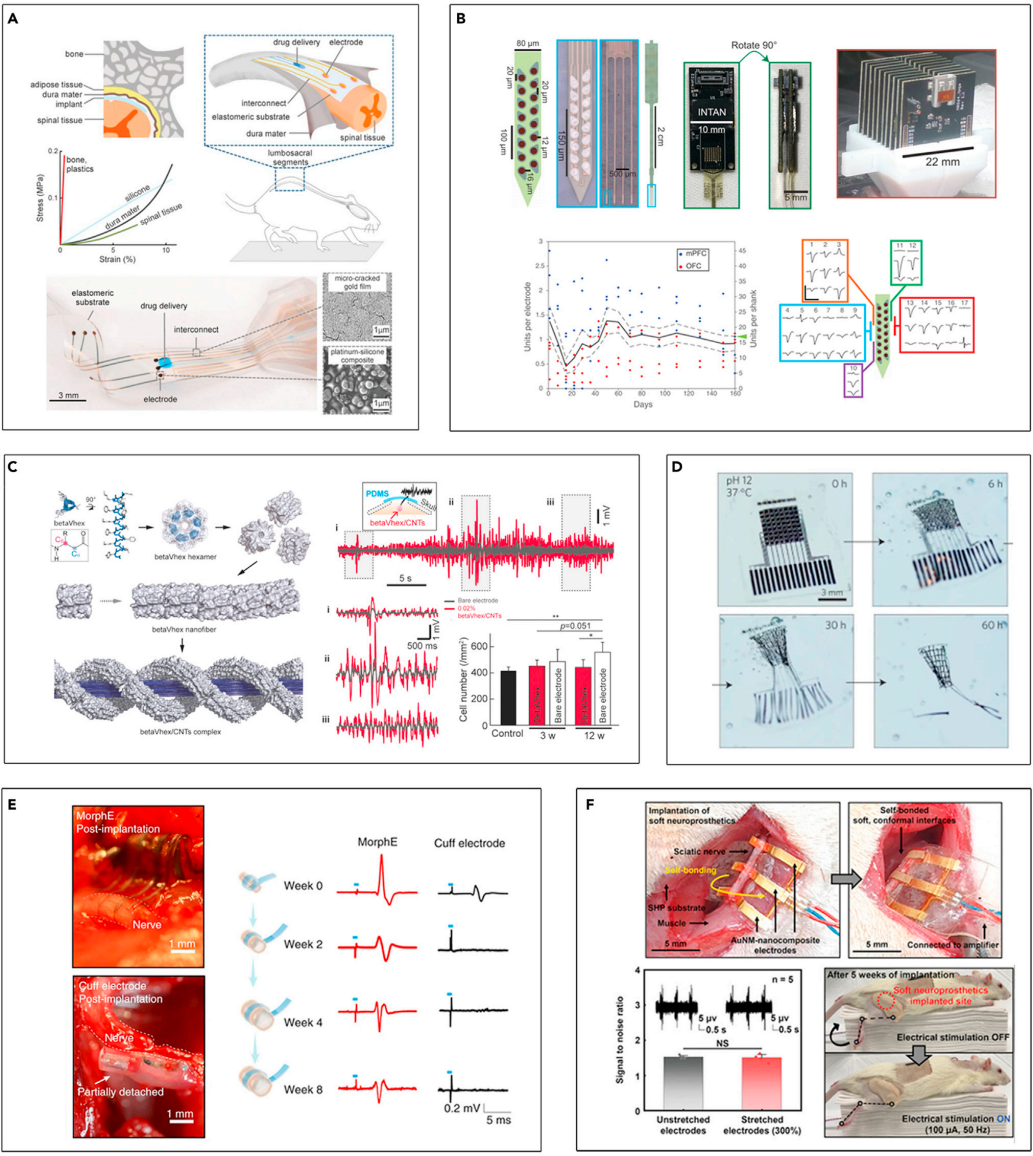
Neural devices based on soft materials (A) Electronic dura mater (e-dura). Reproduced with permission (Minev et al., 2015), Copyright, AAAS. (B) All-polymeric electrode arrays with 1,024 channels and their single-unit recording yield over time. Reproduced with permission (Chung et al., 2019), Copyright, Cell Press. (C) Supramolecular peptide hydrogel-based soft neural interface and the LFP recording signals. Reproduced with permission (Nam et al., 2020), Copyright, American Chemical Society. (D) Bioresorbable actively multiplexed neural electrode array. Reproduced with permission (Yu et al., 2016), Copyright, Springer Nature. (E) Images and representative trace of elicited compound action potentials of cuff electrode and MorphE. Reproduced with permission (Liu et al., 2020), Copyright, Springer Nature. (F) Implantation of fatigue-resistant soft peripheral neural interfacing. Reproduced with permission (Seo et al., 2021), Copyright, Wiley-VCH.

Beyond conventional materials used for neural interfaces exhibiting high stiffness in the range of 1–100 GPa, soft materials such as polymer, elastomers, and gels are being introduced owing to their intrinsic softness, especially those with Young's moduli that are comparable to that of the host tissue (10 kPa–1 MPa). Improved mechanical compliance offers some distinct advantages: (1) long-term use of devices become possible from reduced mechanical mismatch and mitigation of the immune responses (Fallegger et al., 2020), and (2) effective interfacing with 3D convoluted surfaces of tissues for enhanced conformability make the obtainment of high fidelity signals possible. The electronic dura mater (e-Dura), which is a multimodal neural interface that resembles the shape and mechanical property of the dura mater, is one of the representative examples (Figure 2A). It was demonstrated that soft neural implants based on platinum-silicone composites enabled long-term bio integration and functionality within the central nervous system (Minev et al., 2015). As such, soft materials are being actively used in the form of substrates or encapsulation, playing a role as thin film dielectric layers that hold the functional internal components while decreasing any adverse effects from mechanical mismatch issues (Altuna et al., 2012; Araki et al., 2019; Hassler et al., 2011; Luan et al., 2017; Park et al., 2015a; Tybrandt et al., 2018; Viventi et al., 2011; Yan et al., 2017).

Along with the advances in materials engineering, the enhancement of the functional properties of existing materials and the exploitation of emerging materials have opened up a wide range of opportunities to develop much softer neural devices. For example, all-polymeric probes have been reported employing conducting polymers such as conductive carbon-loaded polyethylene (cPE), poly(3,4-ethylene dioxythiophene)-poly(styrene sulfonate) (PEDOT:PSS), and polypyrrole (PPy) as well as carbon-based nanomaterials (Chung et al., 2019; Ferlauto et al., 2021; Khodagholy et al., 2016; Park et al., 2017; Qi et al., 2017). As expected, further softened devices entirely made up of polymers considerably extend the operational lifetime of implants (Ferlauto et al., 2021). Magnetic resonance imaging (MRI) compatibility is another appealing point that all-polymeric devices can offer. Even densely organized polymer electrode arrays based on PEDOT:PSS with 1,024 channels were presented, proving the capability of measuring single unit activities of hundreds of neurons for a prolonged period (>5 months) in freely moving animals (Figure 2B) (Chung et al., 2019). Moreover, there have been emerging approaches to fabricate organic materials-based transistors (Garcia-Cortadella et al., 2020, 2021; Rivnay et al., 2018; Spanu et al., 2021). In particular, the organic electrochemical transistor (OECT) has been investigated as a promising active transducer for its intrinsic amplification capability with tissue-compliant nature (Khodagholy et al., 2013; Rivnay et al., 2018). Based on the mixed ionic/electronic conduction mechanism, conducting polymers like PEDOT:PSS and PPy are typically exploited for the channel of OECTs (Rivnay et al., 2016; Tu and Fabiano, 2020), which can offer several advantages including low operation voltage (<1 V), high transconductance (∼1 mS), and biocompatibility (Khodagholy et al., 2013; Rivnay et al., 2015). A recent study has presented fast, conformable, and implantable OECT made of PEDOT:PSS composite. *In vivo* recording of neuronal action potentials as well as real-time detection of epileptic discharges have demonstrated its capability of high-quality electrophysiological signal acquisition across a broad range of frequencies and amplitudes (Cea et al., 2020). In addition, combined with the evolution of manufacturing techniques such as printing, the rapid prototyping of soft implantable electrode arrays based on polymeric and elastomeric materials becomes possible, which would accelerate the development of versatile neural platforms (Afanasenkau et al., 2020).

More recently, hydrogel-based neural interfaces have drawn extensive interest owing to their intrinsic softness and chemical compatibility to biological tissue (Choi et al., 2021; Huang et al., 2018; Liu et al., 2019; Yuk et al., 2019). They can be utilized for some applications including soft coating agents and adaptive interfaces that can facilitate the implantation by dynamically shifting their moduli upon swelling. As such, based on reduced foreign body responses, hydrogel-based systems have shown promise for improving the long-term stability and reliability of current neural platforms (Liu et al., 2019; Park et al., 2021a). A biocompatible and biostable neural interface consisting of β-peptide-based hydrogels has also been introduced (Figure 2C). Combined with soft multihierarchical structures, integration with the carbon nanotubes (CNTs) enables the formation of complex 3D electrical networks, which can facilitate signal transmission with minimal inflammatory responses. It was further validated by both acute and chronic intercortical and epidural neural recordings, eliciting amplified signals with increased sensitivity (Nam et al., 2020).

Not only leveraging the mechanical properties but additional functionalities of soft materials can also be exploited. Implantable neural interfaces consisting of bioresorbable materials are one of the intriguing approaches (Kang et al., 2016; Xu et al., 2019; Yu et al., 2016). Via hydrolysis and metabolic action, the constituent materials naturally resorb after their programmed period (Figure 2D). Of particular usage, the integration with a stiff biodegradable polymer is a well-known strategy that can effectively handle the buckling issues during device implantation (Lecomte et al., 2018). Provided by enhanced mechanical strength in a transient period, the insertion of soft implantable devices can be facilitated. In addition, because minimal and harmless traces are left, it is an appealing strategy to eliminate the potential risk related to manual removal and reduce the chronic immune responses. Mechanically adaptive neural platforms for recording the peripheral nerve signals are also demonstrated. Typical nerve cuff devices with fixed dimensions usually induce inflammatory issues because of the compressive stresses that are continuously exerted on the growing neural system. To address this limitation, morphing or adaptive peripheral neural platforms that can accommodate the growths of deformations of nerves have been developed based on the viscoplastic elastomer and self-healing polymers (Liu et al., 2020; Seo et al., 2021; Song et al., 2020). Under the compressive stress-free conditions, the reconfigurable interfaces can actively adapt to the growth of nerve tissues *in vivo*, which grows 2.4-fold in diameter with a chronic operation for 2 months (Figure 2E) (Liu et al., 2020). Assisted by the materials' dynamic self-locking properties, no additional fixation methods are needed for interfacing, demonstrating its feasibility for chronic bidirectional signaling (Figure 2F) (Seo et al., 2021).

### Structural innovations for flexible and conformable neural interfaces

Even if the inherent material property is rigid and brittle, the flexibility and conformability of the devices can be achieved by adopting particular structures or geometrical configurations.

**Figure 3:**
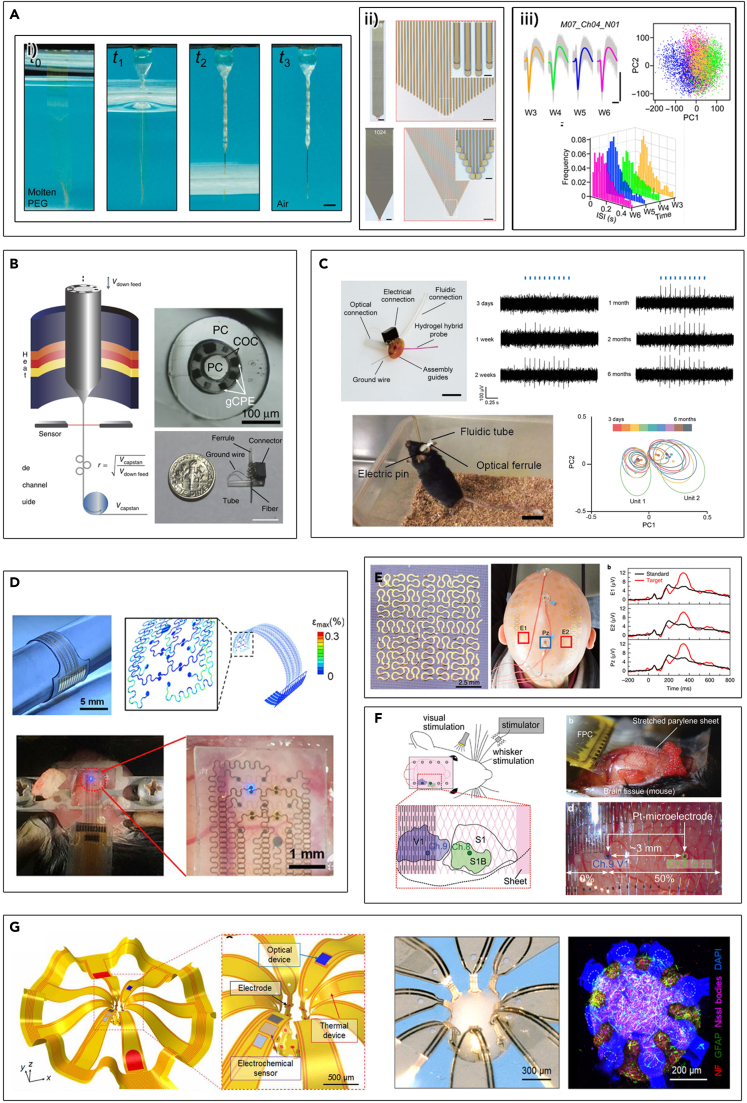
Structural innovations for flexible and conformable neural interfaces (A) (i) Elastocapillary self-assembly of a Neurotassel; (ii) 1024-channel and zoom-in view of the microelectrode filaments; and (iii) chronic stability. Reproduced with permission (Guan et al., 2019), Copyright, AAAS. (B) Multimodal fiber produced by thermal drawing. Reproduced with permission (Park et al., 2017), Copyright, Springer Nature. (C) Adaptive and multifunctional hydrogel hybrid probes and their chronic stability. Reproduced with permission (Park et al., 2021a), Copyright, Springer Nature. (D) Stretchable opto-electric integrated neural interfaces. Reproduced with permission (Ji et al., 2020), Copyright, Elsevier 2020. (E) Serpentine mesh epidermal electronic interfaces for multichannel EEG. Reproduced with permission (Tian et al., 2019), Copyright, Springer Nature. (F) Kirigami-based highly stretchable and flexible bioprobes. Reproduced with permission (Morikawa et al., 2018), Copyright, Wiley-VCH. (G) Microfabricated 3D frameworks for cortical spheroids. Reproduced with permission (Park et al., 2021b), Copyright, AAAS.

One of the characteristic indices used to describe the flexibility of the devices is the bending stiffness, which can be defined as a cubic function of the dimension of the device. Considering that the flexibility can be tailored by the overall size or thickness of the given system, miniaturization is a simple yet effective strategy to manipulate the mechanical compliance and reduce the acute and chronic immune responses (Guan et al., 2019; Guitchounts et al., 2013; Kozai et al., 2012; Park et al., 2015a). Complemented with the progress in micro- and nano-fabrication technology, the neural interfaces with higher functional density in smaller dimensions can be fabricated in a scalable manner. Micro-electrode arrays (MEAs), which are grids of tightly spaced electrodes, are the typical example of the ultrasmall-sized neural platform that can offer the combined advantages of high accuracy, chronic stability, and reliable performances (Didier et al., 2020; Guitchounts et al., 2013; Jun et al., 2017; Kozai et al., 2012; Lee and Someya, 2019; Tybrandt et al., 2018). The densely organized arrays consisting of flexible microelectrode filaments called Neurotassels are representative of MEAs (Figure 3A) (Guan et al., 2019). Each filament holds neurite-scale dimensions, which can be readily assembled to be scaled up to 1024-channel electrodes. Owing to the size reduction, the implanted Neurotassels elicited stable recording over extended periods. Similarly, for active MEAs containing several electronic components such as field-effect transistors or integrated circuits, thickness reduction is an effective strategy to endow flexibility. In this case, ultrathin (∼few μm) active layers made of silicon nanomembrane (SiNM) have been extensively investigated (Fang et al., 2017; Viventi et al., 2011). As an example, Neural Matrix, which is a recently developed flexible, actively multiplexed electrode array for micro-electrocorticography (μ-ECoG), has exhibited remarkable progress as a long-term implantable device with high resolution and scalability (28 × 36 electrodes) with ultrathin (<30 μm) electrode arrays (Chiang et al., 2020). From stable *in vivo* neural signals with high throughputs in rodents and nonhuman primates, it has shown great promise for flexible neural device platforms for prosthetic applications.

Likewise, the introduction of novel form factors is an attractive approach to address current limitations. Thin-fiber-based neural platforms have shown their unique advantages such as high aspect ratio and reduced mechanical mismatch between biotic-abiotic interface (Canales et al., 2015; Park et al., 2019). Especially, the emergence of polymeric composite fibers with predefined architecture fabricated by a thermal drawing process paves a new way for achieving multimodal neural interrogations in a straightforward manner. Via thermal drawing, a multifunctional fiber neural probe made of polymers (diameters <200 μm) has incorporated an optical waveguide, 6 electrodes, and 2 microfluidic channels (Figure 3B). Owing to the flexibility and consequently reduced device footprints, the integrated modalities are successfully demonstrated by chronic interrogation of brain circuits. It was also observed that stable recordings of isolated single-neuron activities persist for 3 months after implantation (Park et al., 2017). Following this context, integration of the polymeric fibers within a soft hydrogel matrix can improve the chemo-mechanical compliance even further, while maintaining multifunctionality (Tabet et al., 2021). Owing to the combined effects of the fibrous form factor and soft material, foreign body responses are minimized so that the hybrid fiber devices allow robust bidirectional modalities that can even track the single neuron activities in freely moving mice for over 6 months (Figure 3C) (Park et al., 2021a).

Construction of geometrical features is one of the strategies to accommodate dynamic deformations while maintaining functionalities (Araki et al., 2019; Heo et al., 2021; Kim et al., 2015; Yan et al., 2017). The principle of the stretchable nature of patterned geometries lies in continuum mechanics, and diverse mesh structures that can effectively dissipate the strain energies can be modeled (Lu and Yang, 2015). Popular examples are as follows: wavy (Qi et al., 2017), serpentine (Fan et al., 2014; Ji et al., 2020; Tian et al., 2019; Wang et al., 2020), honeycomb (Kim et al., 2019; Lee et al., 2018c) structures. Stretchable optoelectric integrated neural interfaces for ECoG have also been developed using similar techniques (Ji et al., 2020). Based on the high flexibility provided by incorporating serpentine-shaped electrodes and multiple micro-light-emitting diodes (μ-LEDs) onto the elastomeric substrates, these surface-type devices have demonstrated *in vivo* optogenetic activation and recording simultaneously while maintaining good performance even after cyclic stretching (Figure 3D). Complemented with the ultra-thin or sacrificial substrates, enhanced conformability is also beneficial in terms of the high-fidelity device performance. Indeed, the electroencephalogram (EEG) tattoo electrodes with serpentine patterns on the scalp have been proposed (Figure 3E). Subsequent EEG data present high-quality signal acquirement, revealing its efficacy attributed to the minimized contact impedance and improved SNR from intimate contact with the convoluted surface structure (Tian et al., 2019). In addition, more complex architectures such as kirigami-inspired structures have been explored for their capability of effectively interfacing with versatile 3D topologies (Morikawa et al., 2018; Shyu et al., 2015; Tang and Yin, 2017). Although the kirigami-based planar electrode usually consists of rather stiff materials within the layered structure, this class of special shapes offers exceptional stretchability, desired for easily tuning the electrode gap over the brain while withstanding the large and repetitive deformations (Figure 3F) (Morikawa et al., 2018).

As a step forward, some recent works have paid attention to manufacturing the volumetric bio-interfaces with organoids or spheroids for fundamental researches and clinical purposes. One of the prominent examples includes the concept called cyborg organoid (Li et al., 2019). Inspired by organogenesis, the *in vivo* 2D-to-3D tissue reconfiguration process, planar mesh nanoelectronics are folded into 3D assembly across the entire organoid while allowing differentiations of stem cells within the embedded nanoelectronic arrays into targeted kinds of functional cells. Minimally invasive and uniformly distributed integration permits the chronic monitoring of electrophysiological activities in human cardiac organoids, suggesting that 2D functional arrays can be effectively integrated with many other kinds of organoids to achieve tissue-wide electrophysiology with enhanced spatiotemporal resolution. Moreover, the manufacturing of sophisticatedly designed 3D mesostructures via compressive buckling was demonstrated. Complex architectures of the microfabricated 3D frameworks for neural interfaces to spheroids with distinct physical features are shown (Figure 3G). With the facile mean of bio-integration, versatile functional elements integrated within the assembly enable multimodal engagements such as electrical, optical, chemical, and thermal interfaces, showing great promise for wide applications (Park et al., 2021b).

### Novel architectures for seamless integration with neural tissue

Efforts have been made to design novel architectures that can effectively interact with the neural system. Based on the design criteria for achieving seamless bio-integration of interfaces within the neural tissue and interfaces, architectures involving bio-inspired or minimally invasive structures have been established.

**Figure 4:**
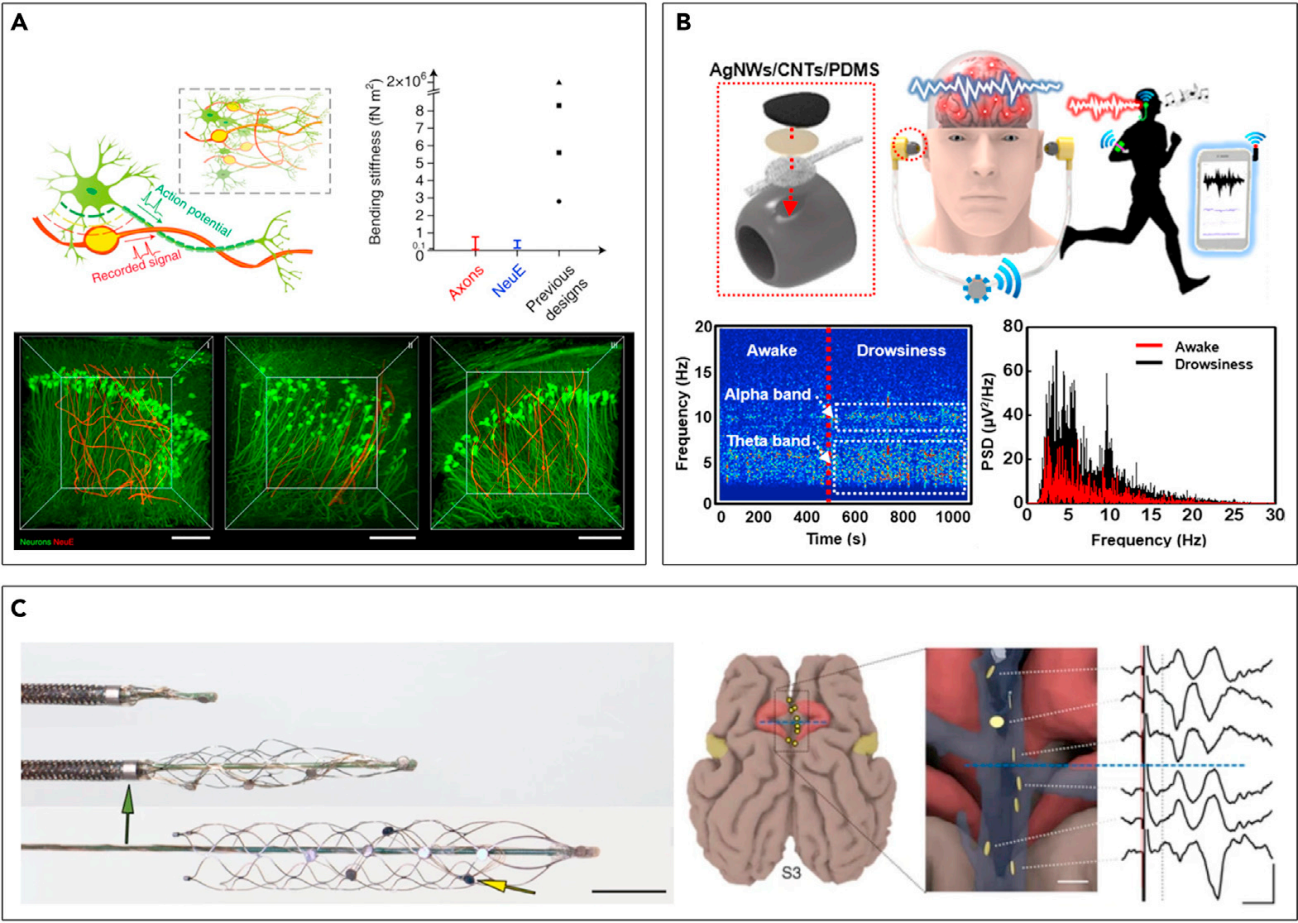
Novel architectures for seamless integration (A) Neuron-like electronics (NeuE) that seamlessly interpenetrate into neuronal networks. Reproduced with permission (Yang et al., 2019), Copyright, Springer Nature. (B) EEG electrodes with personal earphone structure and their recording capability. Reproduced with permission (Lee et al., 2018a), Copyright, American Chemical Society. (C) Minimally invasive endovascular stent-electrode array (Stentrode). Reproduced with permission (Oxley et al., 2016), Copyright, Springer Nature.

Firstly, neural probes inspired by biosystems have been proposed. Based on the structural and mechanical similarity with the brain tissue, the construction of 3D seamless integration within the biological environment can considerably enhance the long-term operational stability. For example, macroporous mesh probes have been proposed, which resemble the neural network (Fu et al., 2016; Liu et al., 2015; Xie et al., 2015; Zhou et al., 2017). Because reduced dimensions and a high degree of porosity (∼80%) offer exceptional flexibility, the open mesh devices permit neurons to form interpenetrating structures with minimal inflammatory responses and effectively interface with the adjacent neurons, just as natural neural tissue sprawled over the brain with intertwined structures. Histology studies exhibited no distinct changes of endogenous distributions of neurons through the mesh probe interior up to 1 year after injection, proving negligible chronic immune responses. Via seamless integration with the biological environment, long-term recording stability at a single neuron level can be also achieved over 8 months (Fu et al., 2016; Zhou et al., 2017). Furthermore, neuron-like electronics called NeuE have been developed (Figure 4A). As its name implies, the probe mimics subcellular feature sizes (∼few μm) and mechanical properties where bending stiffness is comparable to the axons. As brain-dwelling probes, this structural indistinguishability within the microenvironment displays negligible immune responses and demonstrates reliable operations, recording single-unit activities for at least 3 months. Moreover, the intimate interpenetration of newborn neurons was also observed, further suggesting its potential as neural scaffolds (Yang et al., 2019).

As discussed previously, most problems that hinder the long-term and reliable operation of the neural interfaces arise from foreign body responses by invasive surgery. In this context, the practical importance of noninvasive neural interfaces, such as electroencephalogram (EEG), cannot be underestimated. However, conventional EEG systems require bulky electrodes set-up onto the scalp, significantly restricting motion, and prone to noises and artifacts. To address this challenge, Looney et al. proposed an intriguing concept called “in-ear EEG,” monitoring EEG signals from robustly fixed electrodes inside the ear canal (Looney et al., 2012). Although there are some drawbacks such as a limited number of electrodes and reduced signal amplitude, several works have demonstrated its feasibility as a facile and unobtrusive neural interface (Figure 4B) (Hoon Lee et al., 2014; Kidmose et al., 2013; Lee et al., 2018a). Moreover, it was also demonstrated that the signal quality of this dry electrode was comparable to the conventional EEG system using conductive gels owing to the close contact between the devices and sweat/hair-free surfaces (Eickenscheidt et al., 2020; Kappel et al., 2019; Looney et al., 2012).

Utilization of the neurovascular interfaces that can interact with the brain tissues can be another intriguing option. Combined with a well-established cardiovascular technology, this class of device design is highly advantageous in terms of minimal invasiveness and practical efficacy. The most renowned one that has already demonstrated the clinical trials is the Stentrode, the expandable electrode array mounted on the endovascular stent (Figure 4C) (Opie et al., 2017; Oxley et al., 2016, 2021). Several works have proved the feasibility of the technique for monitoring high-fidelity neural signals (Oxley et al., 2016), delivering electrical stimulation (Opie et al., 2018), as well as long-term stability (Opie et al., 2017). Moreover, together with the emerging importance of soft and transient neural interfaces, recently there is an approach to develop bio-degradable neurovascular interfaces (Fanelli et al., 2021). Although it only suggests the possibilities by presenting *in-vitro* analysis, the deployment of neurovascular devices based on slowly degradable polymer and conducting polymer would provide promising results such as improving chronic usability with the aid of soft materials.

**Figure 5:**
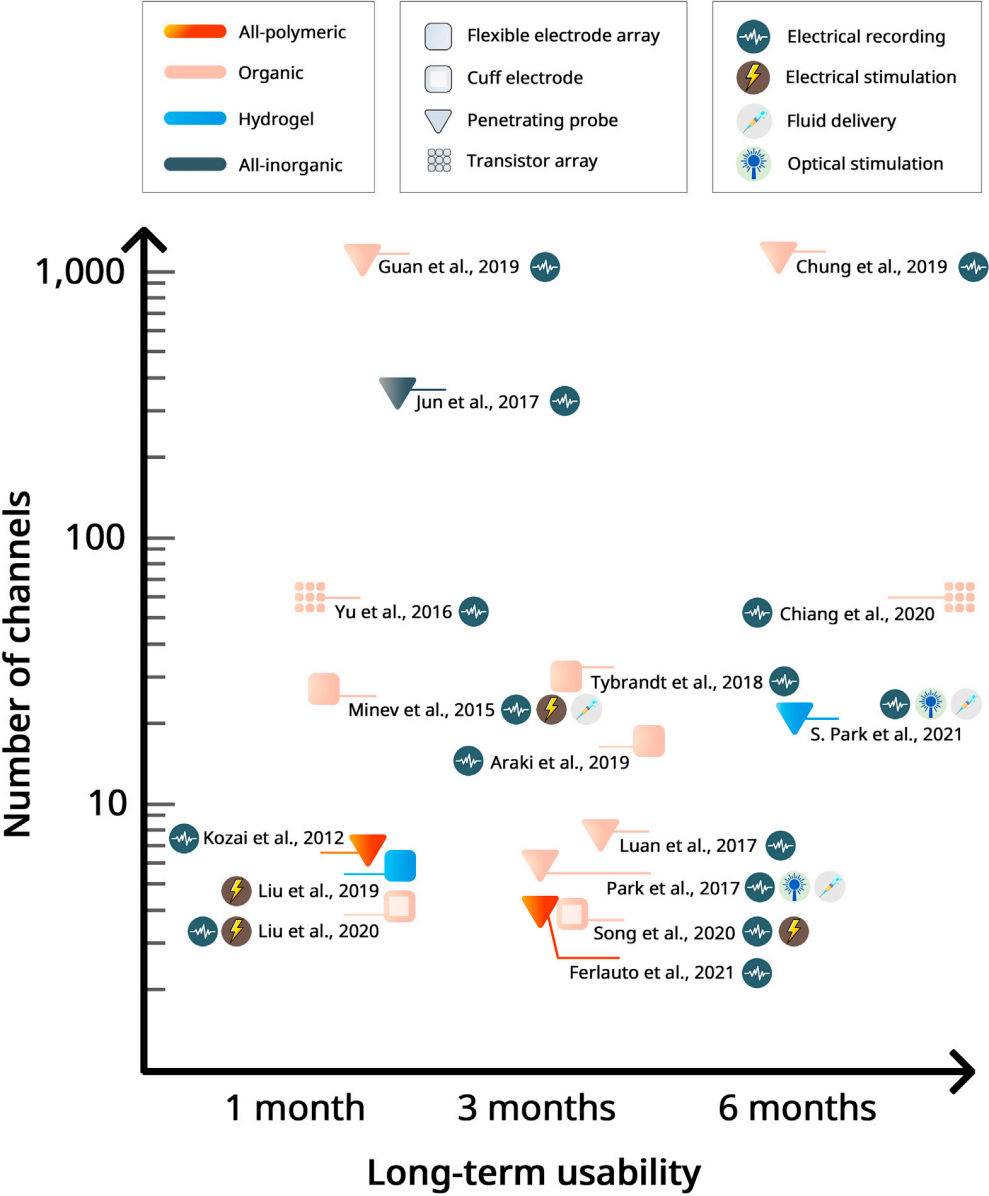
Performance comparison for the typical examples of neural interfaces The x axis represents the long-term usability of the device, and the *y* axis represents the number of recording sites present on the device. The color indicates the key materials utilized to construct the device: all-polymeric, organic, hydrogel, and all-inorganic-based composites. The symbol represents the type of the devices including flexible surface electrode array, cuff electrode, penetrating probe, and transistor array. The icon illustrates different modalities provided by each device. References in alphabetical order (Araki et al., 2019; Chiang et al., 2020; Chung et al., 2019; Ferlauto et al., 2021; Guan et al., 2019; Jun et al., 2017; Kozai et al., 2012; Liu et al., 2019, 2020; Luan et al., 2017; Minev et al., 2015; Park et al., 2017, 2021a; Song et al., 2020; Tybrandt et al., 2018; Yu et al., 2016):

So far, numerous approaches to develop more compliant and feasible neural interfaces have been explored. Some of the discussed examples have been chosen to briefly assess the device performance in terms of the number of channels and long-term usability (Figure 5). In the following section, novel methodologies for achieving high level of sensitivity and cell-type specificity will be addressed.

## Novel methodological approaches in neural recording and modulation

Until recently, neuro-physiological activities have only been recorded through electrical methods with electrodes systems such as EEG, ECoG, and implants. As for stimulation methods, some well-explored modulation schemes include direct electrical stimulation (Kozák and Berényi, 2017) and transcranial magnetic stimulation (Beynel et al., 2020), but recently other methods rely on genetic techniques such as optogenetics (Krook-Magnuson et al., 2013; Ramirez et al., 2013; Yiu et al., 2014) because of its high spatiotemporal resolution and cell-type specificity.

Nonetheless, there are still continuous efforts made in developing novel techniques for neural recording and stimulation, often aspiring to broaden the scope of available tools in neuroscientific studies. In this section, some prominent examples of these novel methodologies will be introduced, and advantages that these techniques bear will be explored in comparison to more conventional schemes.

### New approaches in neural recording methodology

Electrophysiological recording devices have distinct challenges, such as not being cell-type specific, sensitivity being limited by noise acquisition, and increasing invasiveness that comes with high resolution. In this section, two recently developed methods to address these issues utilizing optics and ultrasound transmission will be discussed (Table 1).

**Table 1:** Comparison of conventional and novel neural recording methodologies

	Modality	Advantages	Limitations/Challenges	Reference
Conventional approaches	Electrical	One of the most well-known recording methodologies	Electrical noise and artifact	
	Functional imaging	No disturbance from electrical noise from surroundings	Measure indirect metabolism	
Novel approaches	Neuroplasmonics	No electrical noise, measure direct membrane potential	Low sensitivity	(Kim et al., 2008, 2012; Zhang et al., 2009)
	Ultrasound	The device size can be scaled down to <100 μm	Sensitive to alignment	(Neely et al., 2018; Seo et al., 2016)

#### Plasmonic techniques for neural recording

Electrical noises from surroundings integrated into the signal path have been major obstacles that hinder high-resolution neural recording. Faraday cages or low-noise amplifiers are commonly used to reduce the disrupting effect of noises. Although these techniques can decrease the impact on the SNR and make the problem more manageable, this is not a fundamental solution, and there are limitations to relying on reduction of the noise.

On the other hand, optical methodologies for neural recording are not as prone to such problems because they are not disturbed by electrical noises (Sohrabi and Hamidi, 2016). Some typical examples include functional optical imaging techniques such as fMRI or PET, which takes advantage of intrinsic absorption properties of neuronal cells that vary according to their metabolic activity. However, even though optical signals from these systems are comparatively correlated with the activity of neuronal cells, what they fundamentally measure is an always indirect trace of electrophysiological activities.

**Figure 6:**
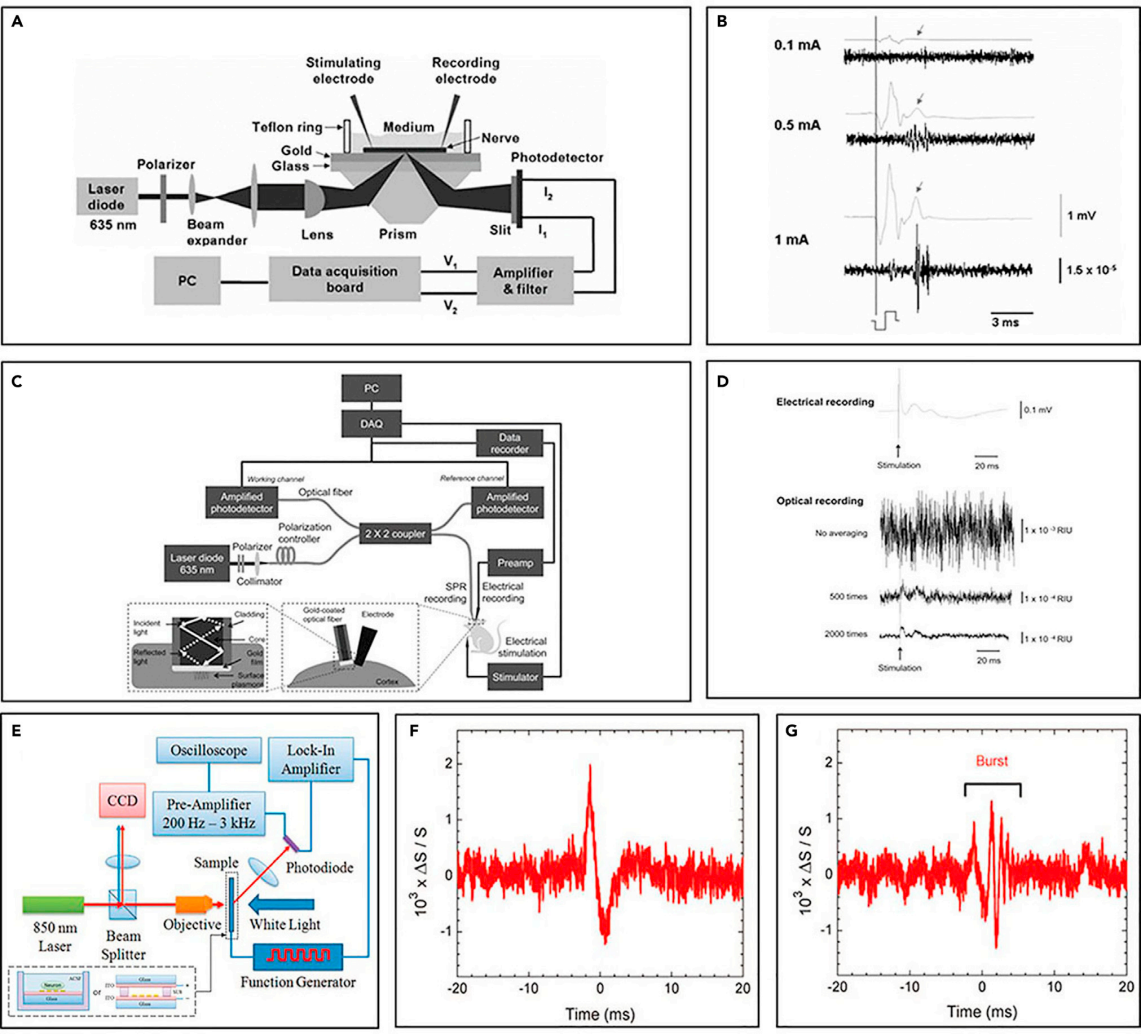
Novel optical techniques for neural recording (A and B) (A) *In vitro* neuroplasmonic setup using gold film and (B) recording results (Kim et al., 2008), Copyright OSA. Electrical signal consists of stimulus activity and relatively smaller neural spikes (arrow), whereas optical signal shows lack of stimulus artifacts. (C) *In-vivo* neuroplasmonic setup using optical fiber with gold film coated on the tip. Reproduced with permission (Kim et al., 2012), Copyright, OSA. Electrical stimulation a forepaw of each rat. (D) The recording result of (C). The optical signal is distinguishable from the noise after averaging 500 cycles (middle trace). (E) The neuronal cells are cultured on the gold nanoparticle array. Reproduced with permission (Zhang et al., 2009), Copyright, American Chemical Society. (F and G) Differential scattering signal measured from neuron-cultured sample, exhibiting a single spike activity (F) and bursting activity (G).

Neuroplasmonics is one of the emerging methodologies recently being studied as a novel approach for directly recording the electrophysiological activity of neurons without the interference of electrical noises. The neuroplasmonics senses surface plasmon resonance to measure the electrical signal based on a shift in the absorbance profile of plasmonic materials such as gold films (Kim et al., 2008, 2012) or gold nanoparticle arrays (Zhang et al., 2009), which is dependent on nearby electrical fields (Figures 6A, 6C, and 6E).

In the neuroplasmonic recording setup, the plasmonic materials are placed close to the target recording site. Typically, the cells are cultured above the plasmonic materials (Figures 6A and 6E) (Kim et al., 2008; Zhang et al., 2008), or plasmonic materials are implanted into the brain (Figure 6C) (Kim et al., 2012). When the light source is illuminated, the light is absorbed through the phenomena known as surface plasmon resonance or, in the case of nano-scale plasmonic materials, localized surface plasmon resonance (Zhang et al., 2009). The amount of absorbed light can then be detected by analyzing the spectrum of the reflected light. If there is electrophysiological activity in the target area, that will apply an electrical field change to the plasmonic materials, which in turn causes the density of electrons to change, resulting in a change in the dielectric constant (Sohrabi and Hamidi, 2016; Zhang et al., 2009). This leads to a shift in the absorbance profile of the plasmonic materials. This little shift of absorbance profile results in significant changes in the amount of absorbed light. By analyzing the absorbance spectrum of the reflected light, the amplitude of electrical field in the recording volume can be calculated (Figures 6B, 6D, 6F, and 6G). As the shift of absorbance profile is closely related to the intensity of the applied electrical field, the direct change in electrophysiological activity, rather than the indirect method of looking for metabolism markers, can be detected in this way. Also, as the signal is recorded and transmitted via optical pathways, electrical noises including stimulation artifacts can be neglected (Figure 6B) (Kim et al., 2008).

#### Ultrasonic techniques for neural recording

**Figure 7:**
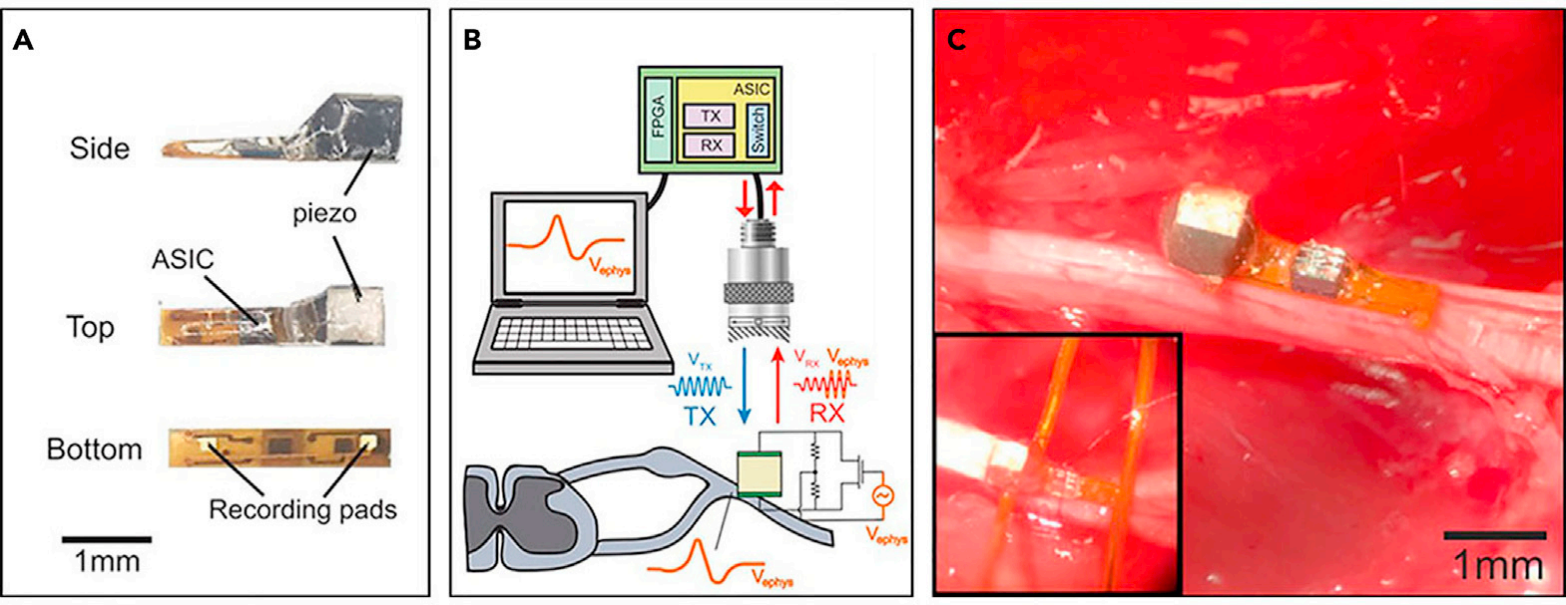
Novel neural recording technique using ultrasound (A) The images of a neural dust mote. Reproduced with permission (Seo et al., 2016), Copyright, Elsevier. A mote consists of flexible PCB, piezoelectric crystal, application-specific integrated chip (ASIC), and a pair of recording pads. (B) The overall experiment setup. The piezoelectric crystal in the mote receives ultrasound energy into electricity, powering the ASIC. The electrophysiological signal is detected between recording pads and modulates backscattered ultrasound signal. (C) The images of neural dust mote anchored on a sciatic nerve of a rat. Inset shows neural dust mote with optional testing leads (inset).

Ultrasound has been highlighted as a novel methodology for its deep penetration depth compared with other modalities such as optical stimulation (Chen et al., 2017). Currently, the application of ultrasound-based recording has typically been limited to structural or functional imaging techniques. As the transducer transmits ultrasound into the tissue, a portion of it is reflected back at the interfaces of different environments that have inconsistent acoustic impedance. The timing and intensity of the reflected ultrasound signals encode the location and density of the structure inside the body. This back-scattered ultrasound, however, can encode electrophysiological signals as well. Seo and his colleagues managed to encode the electrophysiological signal in ultrasound with the aid of a piezoelectric crystal-embedded integrated chip (IC) (Figure 7A) (Seo et al., 2016).

The Neural dust, a mm-scale device equipped with piezoelectric crystal and application-specific integrated circuit (ASIC) for electrical recording, together with an external ultrasonic transducer, comprises the ultrasonic-based wireless peripheral nerve recording system (Figure 7B). When the external transducer exerts ultrasound to the device, some portion of the ultrasound energy is reflected back, whereas some are absorbed in a piezoelectric crystal of neural dust. This absorbed energy generates electricity powering the custom IC for electrophysiological recording. When the electrical signal is recorded between the two electrodes in the IC, it alters the vibration of the piezoelectric crystal. As a result, the backscattered ultrasound signal encodes the electrophysiological signal. Using ultrasound as a carrier of power and communication medium, the researchers could achieve the wireless, closed-loop, fully implanted device (Figures 7B and 7C) for electromyography (EMG) and electroneurography (ENG).

### New approaches in neural modulation methodology

Beyond electrical methodologies, optogenetics enabled researchers to rapidly and precisely modulate specific types of neuronal cells. However, it has been challenging to deliver stimuli to deep regions of the brain noninvasively. Moreover, there have been critics against genetic modification technology itself, as they are considered imperative and inappropriate for clinical usage. In this section, recently developed approaches in neuromodulation technologies that are possible solutions to these challenges will be introduced (Table 2).

**Table 2:** Genetic and nongenetic neuromodulation technologies

	Modality	Related ion channel	Feature	Reference
Genetic modulations	Red light	ChRmine	Optogenetics without intracranial surgery	(Chen et al., 2021)
	NIR light	C1V1/mVChR1 or ChR2 with lanthanide nanoparticles	Attempt to apply NIR band in optogenetics	(Chen et al., 2018; Hososhima et al., 2015)
	Ultrasound	TRP-4	Target individual neurons to US stimulation	(Ibsen et al., 2015)
	Magnetic	TRPV-1, TRPV-4	Negligible attenuation of stimulation in biological tissue	(Chen et al., 2015; Gregurec et al., 2020)
Non-genetic modulations	Electrical		Spatially specific electrical stimulation	(Grossman et al., 2017)
	Ultrasound	Ca^2+^ and NA + voltage-gated channels	Convert US energy to direct-current output	(Marino et al., 2015)
	Visible light	TRPV1	Excitatory neuromodulation, capable of functionalization	(Carvalho-de-Souza et al., 2015)
	NIR light	TREK1	Inhibitory neuromodulation, mechanism equivocal	(Yoo et al., 2014, 2016, 2019)

#### Neural modulation using other genetic techniques

The fundamental of genetic modification technologies is sensitizing a specific type of neurons to certain stimuli that neurons originally did not respond to for rapid and precise modulation. However, delivering stimuli into deeper regions of the brain has been a major obstacle. Traditionally, invasive neural implants were utilized, which have been problematic due to the foreign body responses resulting in the degradation of long-term performance. To resolve this problem, new approaches for genetic tools that sensitize neurons to modalities that penetrate deeper into the brain have been studied.

**Figure 8:**
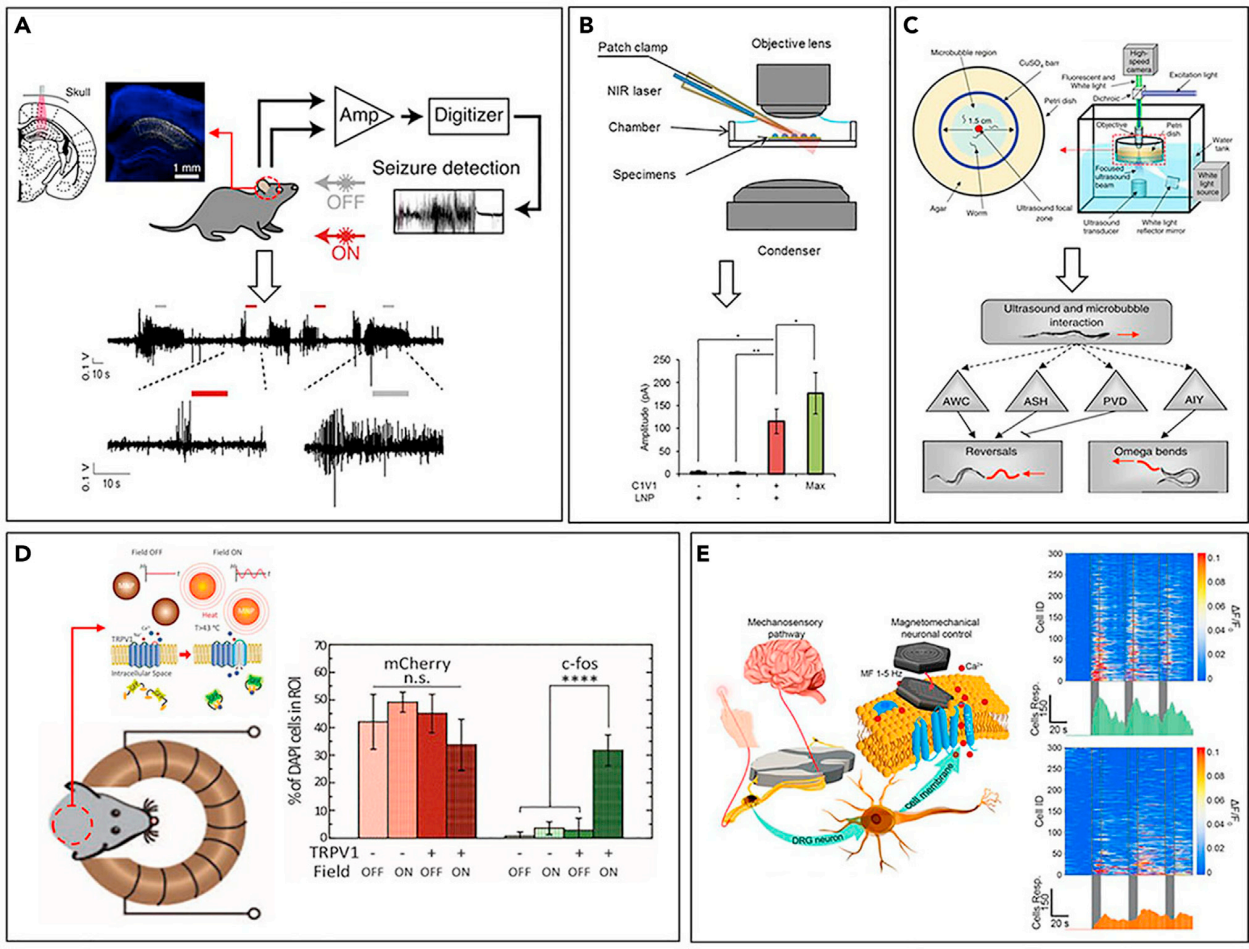
Novel Neuromodulation techniques using genetic modification (A) The experimental setup for closed-loop feedback control for epileptic seizure suppression without intracranial way using ChRmine-yFP (white) expressed at the CA1 hippocampal PV. EEG in the bottom inset shows instant suppression of epileptic seizure upon the optical intervention. Reproduced with permission (Chen et al., 2021), Copyright, Elsevier. (B) Experimental setup for NIR up-conversion optogenetics. The green luminescent light emitted from LNPs effectively activated C1V1 to generate a photocurrent, whereas only a small magnitude of the inward current was generated by the NIR laser light without LNP. Reproduced with permission (Chen et al., 2021), Copyright, Elsevier. (C) The ultrasound is focused onto an agar plate, where animals were corralled into a small area using a copper solution. Sonogenetic neuromodulation of ASH and AWC neurons promote reversals, whereas PVD neurons inhibit reversals. AIY neurons promote omega bends. Reproduced with permission (Hososhima et al., 2015), Copyright, Springer Nature. (D) Magnetic field stimulation induces heat generation of MNP opening TRPV1 channel. The condition where the magnetic stimulation was combined with TRPV-1 expression resulted in significantly large amount of c-fos expression despite the similar level of viral infection. Reproduced with permission (Chen et al., 2015), Copyright, AAAS. (E) Slowly varying magnetic field causes transition of magnetization in the magnetic nanodisc (MND). When the magnetization of MNDs transits from vortex to in-plane, MNDs produce forces on TRPV4 channel resulting in influx of calcium ion and neuronal activation. Reproduced with permission (Gregurec et al., 2020), Copyright, American Chemical Society.

One way to improve the efficiency of light delivery into the deep brain region is to modify the channelrhodopsin so that it can respond to light with deeper penetration depth. A recent study reported a new variant of channelrhodopsin that responds to red light with an extremely large photocurrent that can enter much deeper through brain tissue compared with blue light. This enabled optogenetic modulation of the deep brain region residing up to 7 mm depth, which was shown by targeting the brain stem of a mouse without intracranial surgery (Figure 8A) (Chen et al., 2021).

The penetration depth through biological tissue would be increased further if the scope of available wavelengths was widened to include infrared lights. However, no variant of optogenetic ion channels responsive to NIR light has been developed yet. One way to deal with this problem is by applying genetic modification along with the use of nanoparticles. Nanoparticles can convert energy between modalities. Therefore, one can apply stimuli that can penetrate deeper into the brain and then convert it at the site of stimulation to a modality that ionic channels are sensitized to. A good example of such is the use of up-conversion nanoparticles such as lanthanide nanoparticles that absorb near-infrared light and emit light in the visible spectra, in which most variants of channelrhodopsin are responsive (Figure 8B) (Hososhima et al., 2015).

Ultrasound is also one modality that is renowned for its deep penetration depth. Although the exact mechanism is still being studied, ultrasound has been shown to be capable of modulating neuronal activity. One major problem of using ultrasound stimulation, however, is that high spatial resolution stimulations are limited by the size of the focal point. One recent attempt at resolving this issue was done by expressing TRP-4, the mechanosensitive ion channel to achieve cell-type specificity in modulating neurons of C-elegance. When TRP4 is expressed in SHY, AWC, PVD, or AIY neurons, C-elegans showed significant behavior corresponding to the function of each misexpressed neuron upon low-pressure ultrasound stimulation (Figure 8C) (Ibsen et al., 2015).

Magnetic stimulation exhibits even more penetration depth, its reach being further than any other modality currently used in neuromodulation. Along with its capability in-depth, its high spatiotemporal resolution has made it one of the most promising techniques for neuromodulation. However, the genes encoding ion channels that are sensitized to magnetic stimulation are currently unknown. The magnetic stimulation is therefore commonly used in combination with ion channels that respond to thermal or mechanical stimulation such as TRPV1 (Chen et al., 2015) or TRPV4 (Gregurec et al., 2020) (Figures 8D and 8E). The magnetic nanoparticle serves as a transducer that converts magnetic stimulation into heat (Chen et al., 2015) or mechanical stimulation (Gregurec et al., 2020).

#### Neural modulation using other nongenetic techniques

Genetic modification tools provide powerful tools in neuroscience studies. However, as genetic modification technologies are highly imperative, there is difficulty in clinical translation. For this reason, nongenetic neuromodulation technologies are actively researched as well.

**Figure 9:**
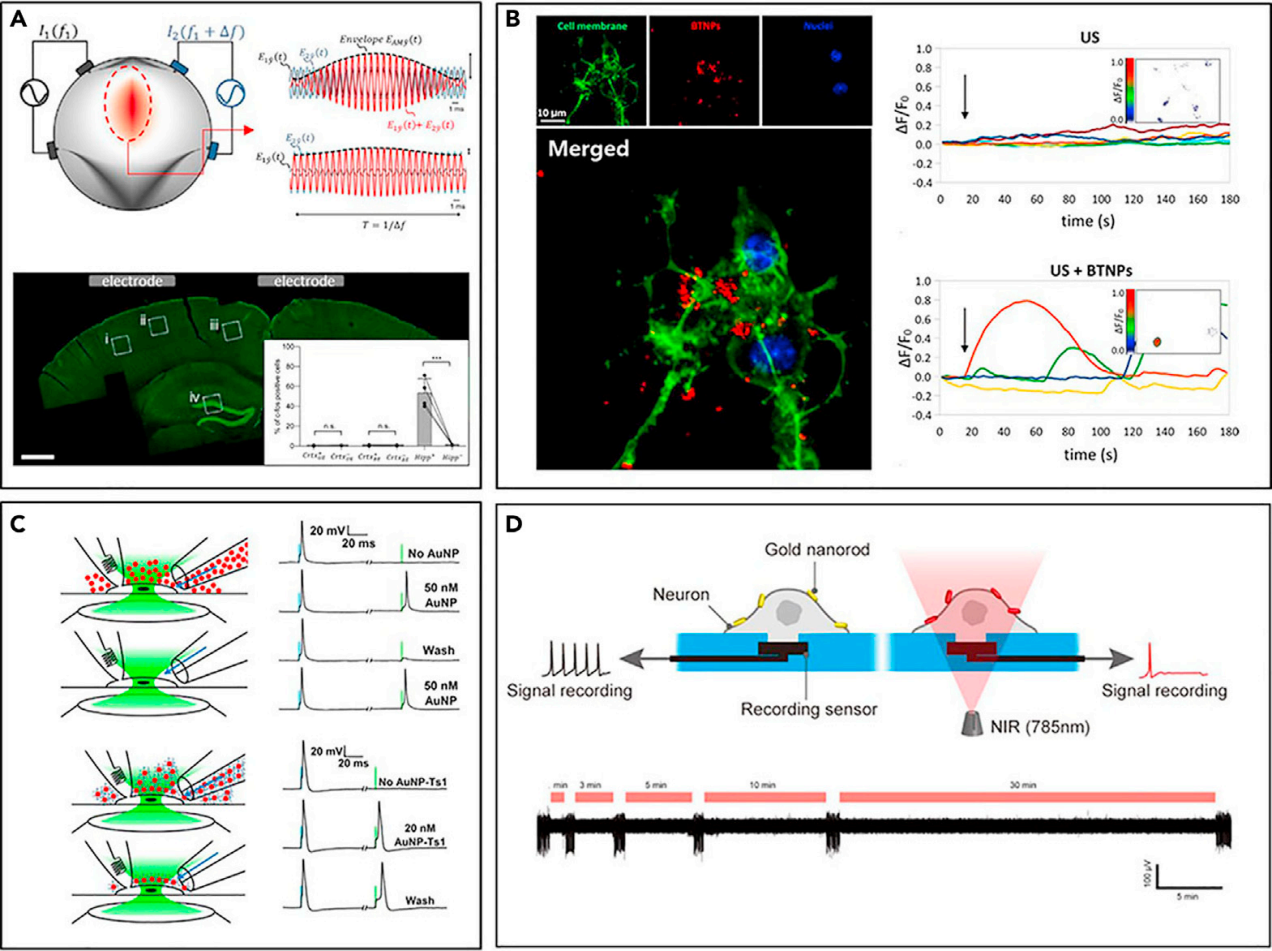
Novel Neuromodulation techniques without genetic modification (A) The center region of colormap represents the sinusoidal electrical field waveforms with the envelope resulting from the superposition of the two fields. Although the c-fos activity was significantly large in the ipsilateral dentate gyrus, rest of the regions showed nonsignificant expression of c-fos as in the bottom inset. Reproduced with permission (Grossman et al., 2017), Copyright, Elsevier. (B) The left inset is confocal fluorescence microscopy of BTNPs attached to the neuronal plasma membranes of SH-SY5Y-derived neurons. (Neuronal plasma membranes in green, BTNPs in red, and nuclei in blue). BTNP combined with US stimulation increased fluorescent signal of calcium imaging. Reproduced with permission (Marino et al., 2015), Copyright, American Chemical Society. (C) GNPs or fresh buffer is perfused over a patch-clamped DRG neuron through either side of a theta capillary (left insets). DRG cells are initially responsive only to the electrical stimulus (blue line), but GNPs sensitized the cells to light stimulation (green light). When GNPs are functionalized, washing does not eliminate optical excitability due to remaining GNPs. Reproduced with permission (Carvalho-de-Souza et al., 2015) Copyright, Elsevier. (D) GNRs coated with NH2-terminated PEG are localized on the neuronal membrane. When the NIR light is illuminated, heat is generated suppressing neural activity. Upon the NIR laser illumination, the activity of cells is inhibited. Reproduced with permission (Yoo et al., 2014), Copyright, American Chemical Society.

One of the most prominent nongenetic neuromodulation technology is simply using electrical stimulation. However, the resolution of electrical stimulation is poor compared with other methods because of the absence in selectivity. A recent study resolved this problem by exploiting the interference between two high-frequency electrical field oscillations, achieving stimulation in deep brain structures such as the hippocampus of a mouse without affecting the cortical regions (Figure 9A, bottom inlet) (Grossman et al., 2017). Although the reactions of various types of neurons are different from the same electrical stimulation, any type of neuron has an upper bound in the frequency of stimulation where it is responsive. When two slightly different, high-frequency electrical fields are applied, temporal interference generates an electrical field oscillating in low frequency that corresponds to the difference of these frequencies, which is localized at the overlapping region. If this difference of frequency is low enough for neurons to follow, only the neurons located in this region will react (Figure 9A, top inlet). By adjusting the ratio of the intensity of the applied electrical field, the overlapping region where the low-frequency oscillations are generated can be shifted. As the neurons outside the stimulation region will be exposed to higher frequency electrical fields that they cannot follow, the spatial specificity of stimulation can be achieved (Grossman et al., 2017).

Another possible technique is applying nanoparticles as interfaces. In contrast to the genetic modification that alters the characteristics of neurons, nanoparticles change the modality of the applied stimuli by playing a role as a nano-scale transducer that converts stimuli that neurons do not respond to into signals that neurons will effectively respond to.

Piezo-nanoparticles such as tetragonal barium titanate nanoparticles (BTNPs) are one of such, in which it converts ultrasonic stimulation into electrical stimulation. In the unit cell of barium titanate, the location of the titanium atom is slightly biased, resulting in an electrical dipole due to asymmetrical charge distribution. BTNP becomes piezoelectric due to this dipole, emitting electrical energy upon the application of mechanical stimulation such as ultrasound. A recent study applied ultrasound on a neuronal culture treated with gum-Arabic-coated BTNP. As the ultrasound stimulation is applied, the gum-Arabic-coated BTNP converts the given ultrasound energy into electrical energy and excites nearby neurons via electrical stimulation (Figure 9B) (Marino et al., 2015).

Gold nanoparticles play a role as nano-scale transducers as well, in which it converts the visible light into heat. The incident electromagnetic wave evokes coherent oscillation of electrons in the conduction band, leading to absorption of the incident light, which is then released in the form of thermal energy. This phenomenon is called the photothermal effect. One useful feature of gold nanoparticles is that the resonance frequency of incident light can be designed by adjusting the diameter or aspect ratio of gold nanoparticles. As mentioned earlier, the NIR band is actively used in biomedical applications, thanks to its deep penetration depth.

The photothermal effect is commonly used in photothermal therapy that induces hyperthermia in cancer cells to eliminate them. However, some recently conducted studies focused on the neuromodulation aspect of the photothermal effect of gold nanoparticles (Figures 9C and 9D) (Carvalho-de-Souza et al., 2015; Yoo et al., 2014). The heat generated by the photothermal effect can be used to excite or inhibit the activity of neurons even though the mechanism of inhibition is still being studied. Sphere-shaped gold nanoparticles can react only in the 500 nm–600 nm band, whereas rod-shaped gold nanoparticles can absorb NIR light, improving biomedical applicability by harnessing the deep penetration depth. Studies done using NIR light and gold nanorods have shown that hippocampal neuron activities can be inhibited using this method (Figure 9D) (Yoo et al., 2014). Another interesting application of gold nanoparticles is to functionalize them with antibodies for cell-targeted neuromodulation. Compared with the neuromodulation techniques using genetic modification, the nongenetic neuromodulation technologies usually lack cell-type specificity. One study took on this challenge by using ligand-conjugated nanoparticles. When chemically biotinylated gold nanoparticles with antibodies against TRPV1 and P2X3 receptors were administered, the proposed gold nanoparticles acquired the ability to optically sensitize specific types of cells as well as display better coupling with membranes (Figure 9C) (Carvalho-de-Souza et al., 2015).

## Progress toward the fully integrated neural system

In many cases, newly developed neural interfaces are only suitable for specific cases within a laboratory environment and are yet to be tried in practical situations. To address this issue, it is crucial that a fully integrated system be devised. This includes considering communications, power supply to the devices, feedback, as well as interactions between nerves and interfaces. In this perspective, recent approaches to the integration of systems with neural interfaces for practical operations are discussed in this chapter.

### Integration of wireless transmission systems

Current neural interfaces are generally wired systems. The tethered connection methods such as wires, optical cables, or fluidic channels mediate the transmission of signals, power, or substance between external sources and targets. However, tethered devices have several downsides such as inhibition of natural motions, large footprints, and risk of external damage. Also, tethered systems cause additional relative movement between the body tissue and the devices, thereby inducing more immune responses. Therefore, usage of these devices in body parts in which the movement is large and frequent is particularly limited. Tethered systems are also reported to inhibit social interaction and induce anxiety behavior (Lu et al., 2018), reducing the applicability in experimental schemes as well as the reliability of the behavioral results.

**Figure 10:**
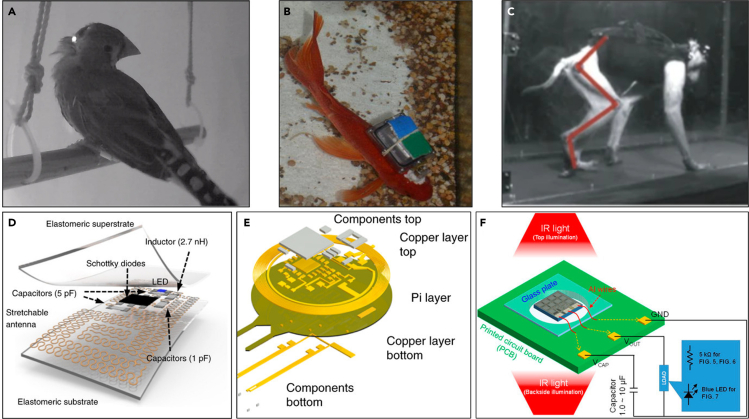
Wireless neural devices (A and B) Examples of various animal models: (A) birds, reproduced with permission (Ausra et al., 2021), Copyright, Springer Nature; (B) fish, reproduced with permission (Cohen et al., 2019), Copyright, MyJoVE Corporation; and (C) nonhuman primates, reproduced with permission (Capogrosso et al., 2016), Copyright, Springer Nature. (D) A fully implantable, soft, and stretchable optoelectronic device operated wirelessly by far-field RF. Reproduced with permission (Park et al., 2015a), Copyright, Springer Nature. (E) A programmable multimodal optogenetic interface using NFC. Reproduced with permission (Gutruf et al., 2018), Copyright, Springer Nature. (F) A 1-mm^3^-sized optical neural stimulator powered by infrared light with CMOS-integrated photovoltaic receivers. Reproduced with permission (Tokuda et al., 2018), Copyright, AIP Publishing LLC.

Integrating wireless systems into neural interfaces aids in avoiding these issues. To begin with, wireless systems have clear advantages in animal experiments in terms of the biological response. These include the reduction of risk of infection in the surgical area, acceleration of recovery, and minimizing the interaction between the device and external environments, which sum up to increasing the overall stability of the system. As other constraints and external effects caused by the presence of tethers are also eliminated, the applicability as well as the reliability of the devices in diverse experiments becomes significantly better as well. For example, the target animal scope can be widened from mice to include more dynamic, larger animals, such as birds (Ausra et al., 2021), fish (Cohen et al., 2019; Wyart et al., 2009), and nonhuman primates (Capogrosso et al., 2016; Zhou et al., 2019) (Figures 10A, 10B, and 10C). Similar to these, the wireless neural interface enables research to be conducted in an environment in which various animals can move freely. It is even possible to target very small animals such as insects (Harrison et al., 2010; Thomas et al., 2012; Zhang et al., 2019b). A recent study introduces a bio-monitoring system using a faster blind-adaptive beamforming technique as compared with conventional methods (Yedavalli et al., 2017) to measure the neural activity or EMG of free-flying insects such as dragonflies (Zhang et al., 2019b).

Wireless systems in neural interfaces are achieved through different modalities such as electromagnetic waves and ultrasound. The most representative method of wireless systems is to control the device and transmit information over electromagnetic waves from the transmitting antenna to the receiving antenna. It is again divided into far-field and near-field methods, depending on the distance between the antennas relative to the wavelength of the electromagnetic wave.

Wireless neural interface using the far-field radio frequency (RF) transmission uses frequencies in the ultrahigh-frequency range (UHF, 300 MHz–3 GHz). It has a relatively long transmission distance up to many meters. Furthermore, this method makes it easier to miniaturize because the size of the antenna required is smaller with higher frequencies. One example of such a device is a fully implantable, soft, and stretchable optoelectronic system that uses a serpentine-shaped antenna that allows capacitive coupling with a rectifying circuit for wireless operation (Figure 10D) (Park et al., 2015a). The antenna covers an area of 3 × 3 mm^2^ at the frequency of 2.34 GHz and performs RF harvesting to supply power to the connected LEDs (a turn-on voltage of 2.7 V, an optical power density of 10 mW/mm^2^) for optogenetic modulation of the spinal cord and peripheral nervous system. Further work using microscale inorganic LEDs (μ-ILEDs) alongside multiple antennas designed with different resonant frequencies even showed individual control of each μ-ILEDs for more sophisticated stimulations (Park et al., 2016). This device was tested in freely behaving mice and showed all remained function for more than 2 months without significant change in operation. Another neural interface, which performs simultaneous measurements at multiple points of the peripheral nervous system provides an example of wireless monitoring (Tang et al., 2017). It records the activity of two separate peripheral nerves with two independent 4-channel cuff electrodes and transmits them to the host computer. The rats used in the experiment were allowed to move freely within the cage, and it showed no abnormalities in functions of the system for 2 months. Here, the telemetry frequency is 3.2–3.8 GHz, and the range of transmission is up to 3 m. However, far-field transmissions have several flaws, of which some important ones are (1) absorption by moisture and body tissue (specific absorption rate, SAR), raising safety concerns that lead to limitation in maximum power; (2) high sensitivity to the angular orientation between antennas; and (3) being prone to the effect of surrounding environmental obstacles.

Wireless systems with near-field transmissions mostly use magnetic resonant coupling, which has a transfer distance of about 1 m. Compared with far-field transmissions, this approach can adopt a relatively simple antenna design and also shows a low absorption rate to tissues as well as being less affected by environmental factors. However, it is also sensitive to the alignment between antennas. The most commonly used frequency is 13.56 MHz, which enables powering, controlling of the device, and data transferring by using near-field communication (NFC) protocols. One case of such a device is a wireless neural cuff system, which is fed power of ∼15 mW from a ∼1-cm-diameter receiver coil through NFC, which is more than sufficient to perform optogenetic and pharmacological neuromodulation to sciatic nerves of a mouse (Zhang et al., 2019a). The operation of the system was demonstrated with freely behaving mice in two weeks after surgical implantation. Optical neuromodulation with μ-ILEDs elevates the temperature, but the increase of temperature (0.85°C with illumination over 5 min) is below the threshold for damage. Another example is a programmable multimodal optogenetic interface that implements a nonvolatile memory of a microcontroller modulated via NFC (Figure 10E) (Gutruf et al., 2018). This device allows users to control the operation parameters wirelessly and digitally, such as stimulation frequencies and duty cycles, by selecting the desired state from the predefined states.

Wireless operation methods through visible or infrared (IR) light have also been studied. In one case, solar cells convert light into power and transmit it to μ-ILEDs to enable optogenetic stimulation (Park et al., 2015b). In another example, a cubic-millimeter-sized optical neural stimulator was developed to acquire power from a complementary metal-oxide-semiconductor (CMOS)-integrated photovoltaic power receiver (Tokuda et al., 2018). It receives infrared light (a peak wavelength of 860 nm) and supplies power to blue LEDs (a peak emission wavelength of 470 nm) (Figure 10F). The optical power conversion efficiency of the device is 4.7% for top illumination. Optical wireless systems can also transmit data over IR communication (Ausra et al., 2021; Burton et al., 2020). An example of such is an optoelectronic neural interface that consists of microscale (250 μm × 57 μm) opto-electrically transduced electrodes (MOTEs) that are powered by visible light (efficiency of 9%, ∼1 μW electrical power consumption) and sometimes return encoded light (near-infrared) to transmit measured neural activity (Lee et al., 2018b).

Ultrasound is an alternative to implementing wireless systems without using electromagnetic waves. As mentioned in the previous section, ultrasound is converted by piezoelectric components, providing power and control signals for the device (Rashidi et al., 2020). Recording devices, such as the Neural dust, can also perform uplink data transmission via backscattering of ultrasound (Johnson et al., 2018; Piech et al., 2020; Seo et al., 2016). Ultrasound has a relatively low absorption rate for biological tissue compared with RF, making it easier to target deeper regions. However, it should be noted that bones exhibit high absorption, and this can cause heat (Nelson et al., 2009). Another downside of ultrasound transmission comes from acoustic impedance matching. Because the impedance mismatch between air and tissues significantly hinders transmission at the boundary of the medium, the transducer usually has to be in close contact with the skin. At the same time, the efficiency of ultrasound is also affected by the alignment of the transmitter and receiver, so it must be correctly positioned.

### Integration methods for a battery-free system

A stable and sufficient power supply is required for long-term and daily use of neural interfaces. In many cases, the required power is supplied using wired connections or batteries. However, tethered systems have a number of problems, as previously addressed, and batteries also have disadvantages. Batteries are the easiest way to replace wires and provide sufficient power for neural interfaces, but their size and weight are significant, accounting for up to 80% of the total device volume (Amar et al., 2015). It hinders the miniaturization of neural interfaces, making them especially difficult to use with implantable interfaces targeting small animals. Apart from such issues rising from increased dimensions, there are safety concerns about the use of batteries, and the presence of batteries, as in wired systems, can affect animal behavior. In addition, replacement or recharging issues are also factors that reduce the reliability of the experiment because they can also impair the natural activity of animals and even directly affect the experiment (Gagnon-Turcotte et al., 2015). Therefore, many studies have been conducted to implement neural interfaces with battery-free systems.

One way to implement a battery-free system is to supply power from an external source using the wireless system discussed in the previous section. Wireless power supplies have the problem of efficiency as well as often being sensitive to positioning such as alignment and distance between external devices and interfaces, which makes it less appropriate for long-term utilization in less-controllable, nonexperimental environments. Therefore, studies are continuing to implement self-powered systems that use internal energy sources such as motions of organs and skeletal muscles, body heat, and biofuel.

**Figure 11:**
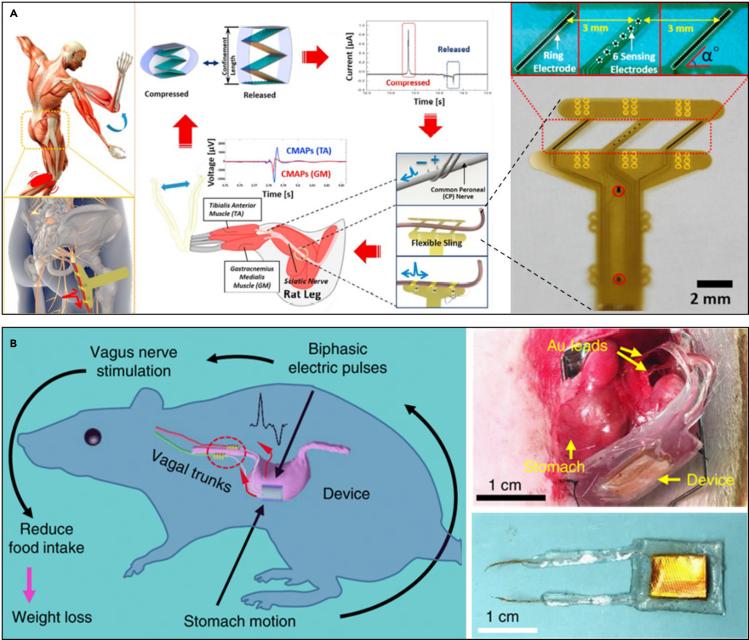
Self-powered battery-free neural interface (A) A battery-free and flexible neural interface based on stacked triboelectric nanogenerators (TENGs) and sling electrodes for direct stimulation of a sciatic nerve. Reproduced with permission (Lee et al., 2017), Copyright, Elsevier. (B) A vagus nerve stimulation system responsive to stomach movement using a flexible and biocompatible nanogenerator. Biphasic electric pulses generated by the movement of the stomach stimulate the vagal afferent fibers and reduce ingestion. Reproduced with permission (Yao et al., 2018), Copyright, Springer Nature.

One method of such power harvesting utilizes piezoelectric or triboelectric materials. Here, piezoelectric and triboelectric transductions are used to convert mechanical energy from voluntary or autonomic muscular motions into electrical energy (Ali et al., 2019; Hwang et al., 2015; Lee et al., 2017; Yao et al., 2018). For example, stacked triboelectric nanogenerators (TENGs) can operate a flexible and adjustable neural interface for stimulation of sciatic nerves and peroneal nerves (Figures 11A). In a specific example, one such case showed a device that has 5 stacked layers connected in parallel, which generates a peak power of 51.8 μW (at the load resistance of 15 MΩ) (Lee et al., 2017). Disadvantages of piezoelectric and triboelectric energy harvesting lie in the low power production and inconsistency of the produced power, usually exhibiting intermittent characteristics. On the other hand, if the energy harvesting pattern relying on the movement pattern of the target location is associated with the intended activity pattern of the device, a device can be implemented that can automatically operate without external control. A device that utilizes such a design principle is one that targets the vagus nerve based on the activity of the gastrointestinal (GI) tract (Yao et al., 2018). Here, biphasic electric pulses are generated in response to the peristalsis of the stomach by a flexible and biocompatible attachment on the surface of the stomach. From the mechanical energy provided by the stomach, the TENG used in the device can generate power up to ∼40 μW at a load of 20 MΩ, which is sufficient to stimulate a nerve. Then, the device stimulates the vagal afferent fibers to regulate intake, and it enables the control of weight (Figures 11B). All rats with the device implantation showed normal daily behaviors, which were no different compared with the control groups. The device achieved 38% weight loss on rats (average body weight of ∼500 g) within 100 days.

Other approaches for battery-free systems using internal sources are the thermoelectric effect and biofuel. Thermoelectric generators provide power using thermal gradients between bio tissues or between the body and adjacent air. When the temperature differential is about 10 K, which is the general difference between air and skin, a wearable thermoelectric generator with flat copper heat spreaders of 1 mm thickness produces only ∼28.5 μW/cm^2^ (Settaluri et al., 2012). Biofuel is a fuel produced from biomass in the body that is converted into electricity through biofuel cells. For example, an implantable glucose biofuel cell produces power from a mammal's body fluids with carbon nanotube/enzyme electrodes that use glucose oxidase for glucose oxidation and laccase for dioxygen reduction (Zebda et al., 2013). It generates power of 161 μW/mL and supplies it to a single LED without causing inflammation in the rat for 110 days. Although the amount of generated energy is still insufficient compared with other methods, these methods show great promise for powering the device by harnessing natural energy sources that are always present in the body.

### Integration with a closed-loop system

Generally, most neural modulations are provided in an open-loop manner (Datta-Chaudhuri, 2021; Sun and Morrell, 2014). However, with the open-loop approach, it is difficult to perform an appropriate modulation according to the subject and its condition. Due to differences between individuals, such as anatomical diversity, the optimal treatment for certain individuals may not be as suitable for others. Even for the same individual, it is not easy to stimulate according to the target's current conditions, because the state of the nervous system or other physiological systems related to the intended modulation is dynamic. On the other hand, a closed-loop system, which can continuously monitor the activities of interest and adequately stimulate with real-time processing, can avoid these problems. In the closed-loop system, more accurate and object-specific treatment is possible by performing real-time adjustments of neuromodulation based on neural activity or other physiological responses.

Closed-loop therapies are also effective for conditional operations that perform a specified role when certain conditions are met, such as relieving pain (Schade, 2011; Schultz, 2012) and treating symptoms of epilepsy (Kassiri et al., 2017; Kozák and Berényi, 2017) as well as Parkinson disease (Gilron et al., 2021; Quinn et al., 2015) by stimulating when pathological phenomena are captured. It also helps the rehabilitation of patients with nerve damage by providing sensory feedback (Ganzer et al., 2020) or by stimulating nerve or muscle stimulation in response to motion commands (Wagner et al., 2018).

**Figure 12:**
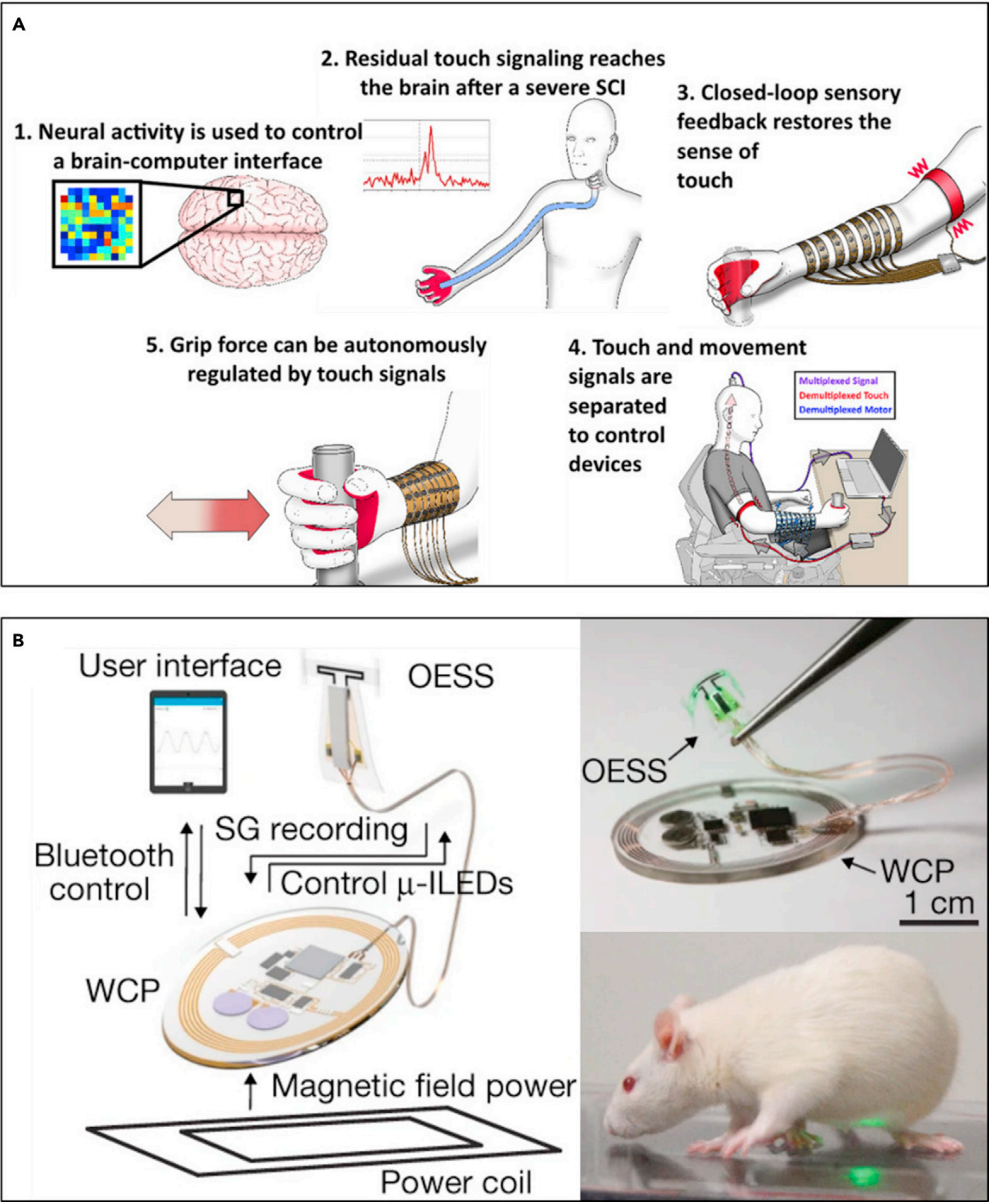
Closed-loop neural interface systems (A) Schematic diagram of a closed-loop demultiplexing BCI for restoring motor function and the sense of touch. Reproduced with permission (Ganzer et al., 2020), Copyright, Elsevier. (B) A fully implantable, wireless, and closed-loop neuromodulation system consisting of wireless control and power (WCP) module, an optoelectronic stimulation and sensing (OESS) module, and a stretchable strain gauge (SG). Reproduced with permission (Mickle et al., 2019), Copyright, Springer Nature.

In some other cases, closed-loop sensory feedback with BCI (Ganzer et al., 2020) is used to restore the sense of touch and motor function of a patient with spinal cord injury. The BCI demultiplexes residual touch signals from the hand and motor signals in the primary motor cortex. Simultaneously, it provides sensory feedback to the bicep and electrical stimulation to the forearm (Figure 12A). This real-time closed-loop sensory feedback enhances sensorimotor function and helps the rehabilitation of the patient. The patients who were equipped with closed-loop sensory feedback achieved over 90% detection rate in the object touch detection task.

Furthermore, studies have recently been conducted to implement a closed-loop system in a form that accompanies the previously addressed wireless or battery-free systems (Cheng et al., 2017; Zhou et al., 2019). This enables more reliable and long-term research or treatment by minimizing restrictions and external intervention on subjects. In one example, a fully implantable, wireless neuromodulation system offers closed-loop operation based on monitoring the bladder to control optical stimulations that regulate pathological physiology (Mickle et al., 2019). The interface consists of a soft and stretchable strain gauge for monitoring bladder filling and voiding, μ-ILEDs (540 nm, about 44 μW emission) for optogenetic peripheral neuromodulation, and a wireless powering and control module (resonance at 13.56 MHz), which allows real-time operation on freely moving animals (Figure 12B). Based on real-time monitoring (a sampling rate of 1 Hz, sufficient for the bladder activity) of bladder function using the soft strain gauge, it identifies the abnormalities of the bladder with the computational method and performs automated optogenetic stimulation using a pair of μ-ILEDs for normalization of bladder function. The devices inserted into rats showed little immune response or other adverse effects, and functioned reliably for over a month.

## Conclusion and perspectives

The studies described earlier present insight into recent novel approaches that are attempting to resolve the challenges of conventionally used neuroscience and neural engineering tools.

One prominent case of those that were dealt with in this paper is that conventional neural probes have suffered from the mechanical mismatch between stiff, brittle materials consisting of the probe and biological tissue. There have been various approaches that tried to minimize these effects. Soft materials contribute to enhancing the long-term performance by matching Young's modulus of a neural probe to that of biological tissue relieving foreign-body response. The modification of the shape or structure of neural probes can improve the conformability and flexibility of devices, as the bending stiffness depends on the dimensions. Also, completely novel architectures such as macroporous probes, Stentrodes, or in-ear EEG systems are being actively developed to improve the interaction between neural probes and biological tissue.

Novel methodology developments in recording and stimulating the neural tissue also broaden the range of available tools that can be used in the field of neuroscience. Although most electrophysiological experiments are conducted via electrical recording or optical imaging, they are yet to be perfected. The electrical pathway that conveys electrophysiological signals easily collects noise from the surroundings. Optical imaging only records the indirect metabolic activity of neuronal cells. Neuroplasmonics has recently gained interest as one of the novel approaches that directly records the electrophysiological activity while neglecting the noise by conveying signals via an optical pathway. As for the stimulation, there are recent approaches of using genetic modifications to deliver proper stimuli to target regions deep in the brain. However, as genetic modification has been criticized to be empirical, there have been advances in nongenetic neuromodulation tools as well, such as nanoparticle-mediated stimuli or temporal interference stimulation.

Although these technologies provide a wide range of new platforms to investigate the functions of the neural system, these approaches were conducted in a specific empirical environment and were difficult to be applied in practical terms. To connect these technological advances to real-life applications, many studies are also working on the integration of diverse systems in neural interfacing devices. The integration of neural interfaces with wireless systems has freed subject animals from tethers and wires, possibly restoring their natural behaviors. Likewise, excluding batteries from the system reduces the dimension and weight of devices not only to restore the natural behavior of subject animals but also to widen the range of available animals for experiments. Integrating the system with a closed-loop system enables precise and adaptable stimulation, which opens the possibility of application in commercial biomedical applications such as rehabilitation and BCI.

As described so far, various remarkable approaches have been conducted in the neural interface field. Thanks to these aforementioned studies, further development of these technologies can be considered. For example, taking advantage of extremely soft materials that have similar elastic modulus with neural tissue would improve the long-term performance of the neural devices. Hydrogel has been renowned for its low Young's modulus, which is almost similar to that of neural tissue. The bending stiffness of neural implants can be reduced by integrating multifunctional sensing and actuation platform within soft hydrogel matrices to ensure adaptive Young's modulus (Park et al., 2021a). Moreover, a multifunctional neural implant made from pure hydrogel may further reduce the bending stiffness to improve long-term performance.

Also, the surface of neural devices can be chemically functionalized and coated with anti-inflammatory materials, which will show similar effects by minimizing foreign body responses. A surface modification technique can modify the surface of implants with a slippery surface inspired by the Nepenthes pitcher plant to achieve excellent repellency against bio-substances (Chae et al., 2020). Inspired by this, the surface of a fiber-based neural interface can be modified to achieve immune evasiveness and therefore reduce the decay of performance caused by immune responses.

In the aspect of methodological and integrating approaches, the plasmonic materials can be coupled with localized light delivery systems. Technologies such as tapered optical fibers (Pisano et al., 2019), tilted fiber Bragg grating (Guo et al., 2017), or holographic photo-stimulation (Yang and Yuste, 2018) can deliver light to a specific location within nanoparticle matrix for spatially specific neuromodulation.

Also, thermally drawn, fiber-shaped batteries have been shown to reduce the bulk and weight of devices significantly in other fields, because this allows the device and its shell to be built out of the battery itself, rather than having a separate portion for the battery (Khudiyev et al., 2020). A similar concept may be possible in neural interfaces because a fiber-shaped battery would significantly decrease the volume the device takes up. These kinds of various approaches are expected to broaden the scope of available methodologies and systems used in neurotechnology and neuroscience as well as pave the way to practically applicable devices.
